# Optimization of Piezoelectric Materials and Ultrasound Imaging Transducers via Alternating Current Poling

**DOI:** 10.3390/s26134292

**Published:** 2026-07-06

**Authors:** Yilei Li, Hao Wang, Ke Zhu, Chenyang Zheng, Jinpeng Ma, Enwei Sun, Xudong Qi, Rui Zhang

**Affiliations:** 1 Functional Materials and Acousto-Optic Instruments Institute, School of Instrumentation Science and Engineering, Harbin Institute of Technology, Harbin 150001, China; 22b901013@stu.hit.edu.cn (Y.L.); 22b901035@stu.hit.edu.cn (H.W.); zhuke@mail.ioa.ac.cn (K.Z.); 21b901031@stu.hit.edu.cn (C.Z.); 20240251@hit.edu.cn (J.M.); sunew@hit.edu.cn (E.S.); 2 Laboratory of Science and Technology on Underwater Acoustic Signal, Institute of Acoustics, Chinese Academy of Sciences, Beijing 100190, China; 3 Key Laboratory for Photonic and Electronic Bandgap Materials, School of Physics and Electronic Engineering, Harbin Normal University, Ministry of Education, Harbin 150025, China

**Keywords:** alternating current poling, domain engineering, piezoelectric materials, relaxor ferroelectric single crystals, piezocomposites, ultrasound imaging transducers, electromechanical properties

## Abstract

Medical ultrasound imaging relies heavily on ultrasound transducers, whose properties directly determine transducer performance. Alternating current poling (ACP) serves as a domain engineering platform to enhance the dielectric and piezoelectric properties of ferroelectric single crystals, ceramics, and piezocomposites compared to conventional direct current poling (DCP). Although several reviews cover the microscopic mechanisms of ACP, researchers have not yet systematically analyzed these materials from the perspective of device applications. This mini-review focuses on the impact of ACP on transducer performance, analyzing the relationship between material properties and device performance in ultrasound imaging transducers. We systematically evaluate the optimization efficacy of ACP across different piezoelectric material forms and bridge the gaps between material parameters and device metrics such as bandwidth and sensitivity. Finally, this review discusses the engineering challenges, structural design synergies, and future trends of ACP-based transducers.

## 1. Introduction

Medical ultrasound imaging serves as a vital tool for clinical diagnosis and intervention, distinguished by its characteristics of a non-invasive nature, real-time capabilities, safety, cost-effectiveness, and portability. Recent advancements propel the field toward functionalization, quantitative diagnosis, and precision medicine. As the core component of the ultrasound imaging system, the ultrasound transducer converts electrical energy into acoustic pulses while functioning as the critical interface with the backend imaging system. The characteristics of the transducer are the primary determinants of critical imaging metrics, including axial and lateral resolution, penetration depth, and signal-to-noise ratio, thereby defining the physical limits of imaging quality. Furthermore, piezoelectric materials act as the primary medium for electromechanical energy conversion within ultrasound transducers. Their intrinsic properties govern the sensitivity, bandwidth, and resolution of the transducers. While the overall imaging and operational metrics of ultrasonic transducers are system-level outcomes heavily dictated by an array of structural and electronic parameters—including acoustic impedance matching layers, heavy backing materials, active element geometries, array aperture design, and driving transceiver electronics—the intrinsic piezoelectric and dielectric properties of the active materials remain a fundamental and central contributor to the final device performance. Consequently, optimizing the performance of piezoelectric materials ensures high-quality medical imaging and advanced diagnostic capabilities.

Currently, high-performance Pb(Zr,Ti)O_3_ (PZT) ceramics and relaxor-PbTiO_3_-based ferroelectric single crystals, such as Pb(Mg_1/3_Nb_2/3_)O_3_-PbTiO_3_ (PMN-PT) and Pb(In_1/2_Nb_1/2_)O_3_-Pb(Mg_1/3_Nb_2/3_)O_3_-PbTiO_3_ (PIN-PMN-PT), have been extensively adopted in medical imaging transducers. Although these materials exhibit superior performance, further enhancing their properties remains a primary research objective to meet the evolving demands of future applications. Traditionally, the dielectric, piezoelectric, and temperature stability of these materials are improved through compositional adjustments, ion doping and the optimization of preparation processes. However, these strategies often incur high research and development costs, significant technical complexity, and long development cycles. In 2014, Yamashita and colleagues introduced an innovative approach known as alternating current poling (ACP), which enhanced the dielectric constant by approximately 50% and the piezoelectric coefficient by 53% compared to standard DCP [1,2,3]. Subsequently, researchers have extensively investigated the ACP method, covering investigations into its formation mechanism, expansion of its application scope, optimization of poling parameters, and the exploration of ACP-based device applications.

Conventional direct current poling (DCP), representing the final stage in the fabrication of piezoelectric materials, has remained largely unchanged for decades. This procedure primarily broke spatial-inversion symmetry and induce macroscopic piezoelectric responses. Since its introduction, ACP has transitioned from a laboratory observation into an effective methodology for modulating the properties of piezoelectric materials. Compared to conventional DCP, ACP utilizes an alternating electric field to induce a finer and more uniform domain structure in single crystals and ceramics, thereby significantly enhancing parameters such as the dielectric constant (*ε*), piezoelectric coefficient (*d*_33_), and electromechanical coupling coefficient (*k*). Current research progress indicates that ACP functions not merely as a specific operation involving “poling procedure using alternating electric field”, but rather as a “domain engineering platform”. This platform is a multi-parameter modulation scheme. It harnesses the tunable waveform, frequency, and amplitude of AC fields to deliberately control domain size, orientation, and distribution. This goes beyond the single-step switching of DCP. Designing the electric field parameters and coupling them with other physical fields achieves multi-dimensional tailoring of the domain structure. ACP is thus positioned as a versatile microstructure-tailoring tool that links material properties directly to specific transducer demands.

Although several excellent review articles on the ACP method have been reported to date, existing reviews have primarily focused on microscopic mechanisms or material-level performance optimization, lacking a systematic analysis from the perspective of device applications [4,5,6,7]. In practice, device implementation ultimately realizes the technical value of ACP. Herein, we focus on the impact of ACP on transducer performance, the relationship between material properties and device performance, and the prospects of ACP in ultrasound imaging transducers. This mini-review aims to systematically summarize the recent progress, provide critical physical insights, and identify potential application opportunities and challenges for alternating current poling (ACP) technology in high-performance medical ultrasonic imaging transducers.

## 2. Performance Optimization of Piezoelectric Materials via the ACP Method

**Figure 1:**
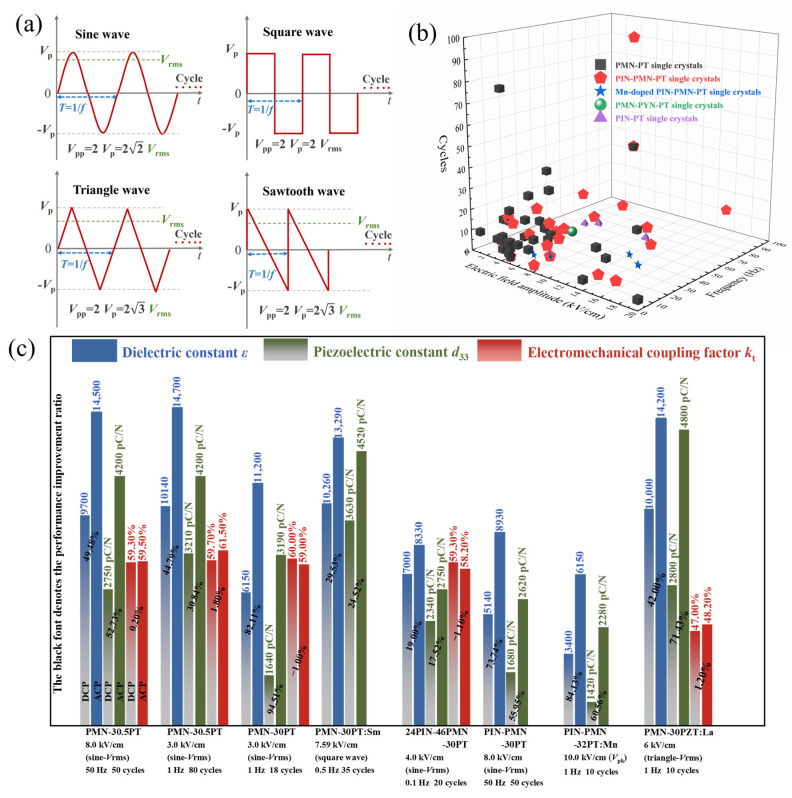
(**a**) Different ACP voltage waveforms [10]; (**b**) Optimal poling conditions for ACP in various relaxor single crystal systems; (**c**) Comparison of dielectric constant *ε*, piezoelectric constant *d*_33_, and electromechanical coupling factor *k*_t_ between DCP and ACP for representative single crystals.

Since the ACP method was proposed in 2014 [1,8], researchers have extensively investigated it across a range of materials, including single crystals, ceramics, and piezoelectric composites. In recent reports, the AC signal waveforms used for ACP are generally classified into four types: sine waves, square waves, triangular waves, and sawtooth waves [9,10]. For specific research systems, the performance impacts of these waveforms exhibit significant differences. Additionally, expressions for the amplitude of the AC electric field vary across different studies, involving peak value (*V*_pk_), peak-to-peak (*V*_pp_) value, and root mean square (*V*_rms_) value. In view of this, we illustrate the waveforms and the conversion methods of various electric field amplitudes in Figure 1a.

### 2.1. ACP Performance Optimization of Relaxor Ferroelectric Single Crystals

Currently, the vast majority of ACP research focuses on relaxor ferroelectric single crystals. Systems that have been proven to achieve performance enhancement through ACP include PMN-PT, PIN-PMN-PT, Mn-doped PIN-PMN-PT, Pb(In_1/2_Nb_1/2_)O_3_-PbTiO_3_ (PIN-PT), Pb(Mg_1/3_Nb_2/3_)O_3_-Pb(Zr,Ti)O_3_ (PMN-PZT), Pb(Zn_1/3_Nb_2/3_)O_3_-PbTiO_3_ (PZN-PT) and Pb(Mg_1/3_Nb_2/3_)O_3_-Pb(Yb_1/2_Nb_1/2_)O_3_-PbTiO_3_ (PMN-PYbN-PT). In addition, there are also reports on the application of ACP to lead-free K(Ta,Nb)O_3_ (KTN)-based and (Na,Bi)TiO_3_ (NBT)-based single crystals [11,12,13,14,15,16,17,18,19,20,21,22,23,24,25,26]. Reported research topics related to ACP include: optimization of poling conditions, microstructural evolution mechanisms, metastable phase, sample thickness dependence, over-poling effects, full matrix parameters, aging behavior, depoling, combination of DCP and ACP, intrinsic and extrinsic responses, coercive field evolution, energy harvesting, and photovoltaic effect applications [27,28,29,30,31,32,33,34]. In previously published reviews, Sun, Kim, Baasandorj, and Maiwa et al. have detailed the relevant research on ACP optimization in relaxor ferroelectric single crystals [4,5,6,7]. Herein, we primarily focus on a systematic statistical analysis, comparing key performance parameters and the maximum achievable performance of Pb-based relaxor single crystals processed by the ACP method. This section aims to provide an intuitive comparison and serve as a reference for the engineering applications of ACP. Tables S1–S4 comprehensively summarize relevant reports on ACP-optimized relaxor ferroelectric single crystals from 2018 to 2025, specifically listing poling conditions and performance comparisons [10,11,13,14,15,16,17,18,19,21,24,26,27,28,29,30,31,32,33,34,35,36,37,38,39,40,41,42,43,44,45,46,47,48,49,50,51,52,53,54,55,56,57,58,59,60,61,62,63,64,65,66,67,68,69,70,71,72,73,74,75,76,77,78,79,80,81,82,83,84,85,86].

It is well established that the ACP process is governed by three controllable variables: poling electric field amplitude, poling frequency, and poling cycles. Figure 1b summarizes the currently reported optimal poling conditions for ACP in various relaxor single crystal systems. When combined with the data in Tables S1–S4, it is evident that the optimal conditions for relaxor single crystals generally fall within the ranges of 1–8 kV/cm (Vpk), 1–50 Hz, and 1–30 cycles. PMN-PT single crystals typically utilize low-frequency poling (≤1 Hz), whereas PIN-PMN-PT single crystals prefer high-frequency poling (≥10 Hz). However, this is only a general trend and is not universally applicable. Indeed, even for samples with identical compositions within the same system, significant variations exist in the optimal poling conditions reported across different studies. These discrepancies can be partially attributed to variations in crystal growth methods and processes, crystal quality and defect density, compositional deviations, crystal dimensions, and the specific ranges of experimental conditions explored. However, a more fundamental reason may be that a single material does not necessarily possess a unique optimal poling condition or a single optimization mechanism.

For instance, 0.695PMN-0.305PT single crystals grown by the same modified Bridgman method achieved identical, exceptionally high piezoelectric properties (*d*_33_ = 4200 pC/N) under completely different ACP conditions (8 kVrms/cm sine wave, 50 Hz, 50 cycles and 3 kVrms/cm sine wave, 1 Hz, 80 cycles) [35]. Although the origin of such conflicting behaviors remains unclear, a common inference can be drawn: significantly different poling conditions induce distinct microscopic domain structures, and despite these structural differences, all can contribute to excellent piezoelectricity. Logically, high-frequency poling combined with a high electric field tends to induce uniform, small-sized domains with high domain density, enhancing the contributions of reversible and irreversible domain wall motion during device operation [17,36]. In contrast, low-frequency poling combined with a low electric field favors the formation of uniform, large-sized domains, which enhances the intrinsic piezoelectric response arising from lattice deformation. Both approaches can achieve a substantial enhancement in piezoelectricity. Currently, the absence of a strict comparative analysis among poling conditions, microstructure, and macroscopic performance makes it difficult to thoroughly resolve these issues. Nevertheless, an important insight has emerged: ACP is not merely a poling process, but a highly promising domain engineering platform.

From the perspective of performance optimization, Figure 1c illustrates representative studies on ACP-optimized relaxor ferroelectric single crystals reported to date. Additional relevant research is summarized in Tables S1–S4. In 2022, Wang et al. achieved an ultrahigh free dielectric permittivity ($\varepsilon_{33}^{T}$/*ε*_0_ = 14,500) and piezoelectric constant (*d*_33_ = 4200 pC/N) in [001]_C_-oriented 0.695PMN-0.305PT single crystals using the ACP method (8.0 kV_rms_/cm sine wave, 50 Hz, 50 cycles) [35]. This value represented the highest reported *d*_33_ at that time, surpassing even the highly representative Sm-doped PMN-PT single crystals (*d*_33_ = 4100 pC/N). Consequently, this work is regarded as a landmark study in the application of the ACP method. Compared to its DCP counterpart ($\varepsilon_{33}^{T}$/*ε*_0_ = 9700 and *d*_33_ = 2750 pC/N), the $\varepsilon_{33}^{T}$/*ε*_0_ and *d*_33_ of ACP-processed 0.695PMN-0.305PT single crystals exhibited improvements of approximately 50% and 53%, respectively [37]. However, no significant improvement was observed in the electromechanical coupling coefficient *k*_t_. Based on XRD analysis, the authors attributed the enhancement in dielectric and piezoelectric properties to the ACP process, which eliminates 71° domain walls and stabilizes 109° domain walls in [001]_C_-poled monoclinic PMN-PT single crystals.

In 2025, Sun et al. employed a low-frequency poling condition (3.0 kV_rms_/cm sine wave, 1 Hz, 80 cycles), which also achieved superior dielectric and piezoelectric properties ($\varepsilon_{33}^{T}$/*ε*_0_ = 14,700 and *d*_33_ = 4200 pC/N) in [001]_C_-oriented 0.695PMN-0.305PT single crystals [38]. As shown in Figure 1c, these parameters represent improvements of approximately 45% in $\varepsilon_{33}^{T}$/*ε*_0_ and 31% in *d*_33_ compared to the DCP-processed counterpart. Furthermore, the low-frequency ACP-processed 0.695PMN-0.305PT single crystals exhibit optimized *k*_t_ and dielectric loss (tan *δ*). Specifically, the *k*_t_ increases by 1.8% from 59.70% to 61.50%, while the tan *δ* decreases by 0.33% from 0.57% to 0.24%. The improved *k*_t_ and decreased tan *δ* are crucial for optimizing transducer performance. SEM microstructures of the 0.695PMN-0.305PT single crystals indicate that the DCP sample possesses a complex domain structure resembling an irregular mosaic pattern. In contrast, ACP samples under optimal poling conditions display distinct, regular, and uniform 109° domain walls. This microstructural evolution is responsible for the improved piezoelectric properties of the PMN-PT single crystals.

The optimal ACP parameters are non-unique. For instance, condition A is 8 kVrms/cm, 50 Hz, and 50 cycles; condition B is 3 kVrms/cm, 1 Hz, and 80 cycles. Both achieve a record-high *d*_33_ ≈ 4200 pC/N in identical single crystals. This reveals the highly complex multi-path nature of the ACP domain engineering platform. Dynamically, the energy state of a ferroelectric system can be driven toward multiple local minima in the Landau free-energy landscape. Low-frequency, low-field ACP drives a slow, near-equilibrium thermodynamic process. This process allows defect boundaries to relax, eliminates high-energy 71° domain walls, and stabilizes large, highly aligned 109° stripe domains. This minimizes elastic clamping and enhances the intrinsic lattice deformation contribution. On the other hand, high-frequency, high-field ACP drives a far-from-equilibrium kinetic process. The rapid voltage oscillations suppress domain wall coarsening, resulting in a highly refined, uniform, and high-density domain pattern. This maximizes the extrinsic domain wall mobility. That both pathways converge on the same macroscopic performance (*d*_33_ = 4200 pC/N) underscores that the ACP process is not a rigid singular procedure, but rather a versatile platform. This platform is capable of accessing different local microstructural states to yield optimized properties.

The abovementioned two studies represent the state-of-the-art performance currently achievable for PMN-PT single crystals using the ACP method. To date, the most significant enhancements in dielectric and piezoelectric properties using the ACP method have been observed in [001]_C_-oriented 0.70PMN-0.30PT single crystals, as reported by Ke et al. in 2025 [39]. As shown in Figure 1c, under the poling condition of 3.0 kV_rms_/cm sine waves at 1 Hz for 18 cycles, the *d*_33_ increases by approximately 95% from 1640 pC/N to 3190 pC/N, while $\varepsilon_{33}^{T}$/*ε*_0_ increases by approximately 82% from 6150 to 11,200. Conversely, *k*_t_ exhibits a slight decrease from 60% to 59%. In addition, Luo et al. (2019) [40] also achieved a significant 67% improvement in both $\varepsilon_{33}^{T}$/*ε*_0_ and *d*_33_ in [001]_C_-oriented 0.70PMN-0.30PT single crystals using a low-frequency ACP method. In that study, the *d*_33_ increases from 1916 pC/N to 3200 pC/N and $\varepsilon_{33}^{T}$/*ε*_0_ increases from 6287 to 10,500, accompanied by reduced dielectric loss. Their findings suggest that a low poling frequency is preferable for PMN-PT single crystals.

For first-generation PMN-PT single crystals, ACP-related research has primarily focused on compositions ranging from PMN-0.25PT to PMN-0.31PT. Performance optimization is predominantly achieved in the [001]_C_ orientation. Specifically, the maximum absolute piezoelectricity achieved by ACP is 4200 pC/N in 0.695PMN-0.305PT single crystals, whereas the maximum proportional improvement in piezoelectric response is 95% in 0.70PMN-0.30PT single crystals. The poling conditions employed across different studies exhibit significant diversity, as summarized in Table S1. Furthermore, the efficacy of ACP in optimizing the electromechanical coupling coefficient *k*_t_ (thickness vibration mode) is limited, which may be a limiting factor for the design of broadband medical imaging transducers.

For second-generation PIN-PMN-PT single crystals, the ACP has also demonstrated effectiveness in performance optimization. To date, more than 30 papers reporting improved dielectric and piezoelectric properties in PIN-PMN-PT single crystals using ACP have been published, as summarized in Table S2. The reported compositions range from PIN-PMN-0.25PT to PIN-PMN-0.41PT. Similar to PMN-PT single crystals, performance optimization of PIN-PMN-PT single crystals is predominantly achieved in the [001]_C_ orientation. However, current reports indicate that PIN-PMN-PT single crystals still lag behind PMN-PT single crystals in terms of both maximum achievable performance and the maximum ratio of performance enhancement. Figure 1c illustrates two representative cases for ACP-optimized PIN-PMN-PT single crystals. Both the maximum achievable performance and the maximum ratio of performance enhancement are observed in [001]_C_-oriented PIN-PMN-30PT single crystals. In 2022, Luo et al. [37] employed field-cooling ACP (4.0 kV_rms_/cm sine wave at 0.1 Hz for 20 cycles from 100 °C to 70 °C) to obtain an enhanced $\varepsilon_{33}^{T}$/*ε*_0_ of 8330 and *d*_33_ of 2750 pC/N in ACP-processed 0.24PIN-0.46PMN-0.30PT single crystals ([001]_C_ orientation). In this case, the *d*_33_ increases by approximately 18%, while $\varepsilon_{33}^{T}$/*ε*_0_ increases by approximately 19% compared to the DCP counterpart. Although the *d*_33_ of 2750 pC/N represents the highest value currently reported for ACP-processed PIN-PMN-PT single crystals, there is a slight decrease (1.10%) in *k*_t_ for ACP samples.

The maximum ratio of performance enhancement in PIN-PMN-PT single crystals using ACP was reported by Wang et al. in 2022 [16]. In this study, ACP induced an approximate 74% increase in $\varepsilon_{33}^{T}$/*ε*_0_ (from 5140 to 8930) and an 56% increase in *d*_33_ (from 1680 pC/N to 2620 pC/N) in [001]_C_-oriented PIN-PMN-0.30PT single crystals compared to the DCP counterpart. The poling condition used was a sine wave of 8.0 kV_rms_/cm at 50 Hz for 50 cycles at 50 °C. In 2020, Ma et al. also achieved significant enhancement in dielectric and piezoelectric properties of [001]_C_-oriented 0.25PIN-0.43PMN-0.32PT single crystals using the ACP method. Under the poling condition of 10.0 kV_pk_/cm at 50 Hz for 20 cycles, the $\varepsilon_{33}^{T}$/*ε*_0_ and *d*_33_ of the ACP-processed samples improve to 7120 and 2610 pC/N, which are approximately 48% and 54% higher than those of the DCP counterpart ($\varepsilon_{33}^{T}$/*ε*_0_ = 4800, *d*_33_ = 1700 pC/N) [25]. In 2025, Jing et al. reported an enhancement in $\varepsilon_{33}^{T}$/*ε*_0_ and *d*_33_ by ACP in [011]_C_-oriented R-phase 0.27PIN-0.46PMN-0.27PT single crystals under the poling condition of 15.0 kV_pk_/cm at 100 Hz for 10 cycles. Here, the $\varepsilon_{33}^{T}$/*ε*_0_ and *d*_33_ of the ACP sample reach 2780 and 676 pC/N, increases of approximately 16% and 6%, respectively, compared to the DCP counterpart ($\varepsilon_{33}^{T}$/*ε*_0_ = 2394, *d*_33_ = 637 pC/N). Although the degree of improvement is limited, this study indicates that ACP can also be used to optimize the performance of “2R”-engineering-domain relaxor ferroelectric single crystals [36].

Feng et al. investigated the effects of ACP on single crystals of 23PIN-47PMN-30PT with different orientations in 2025. Poling conditions of 10 kV/cm and 1 Hz were applied for 25 cycles to single crystals oriented along [001], [011], and [111]. Compared to DCP samples, ACP increased *d*_33_ and $\varepsilon_{33}^{T}$/*ε* by 18.03% and 33.26%, respectively, in the [001]-oriented PIN-PMN-PT single crystal, but decreased *d*_33_ and $\varepsilon_{33}^{T}$/*ε*_0_ by 5.66% and 8.86% in the [011]-oriented crystal. The *d*_33_ value of the [111]-oriented PIN-PMN-PT single crystal under ACP was only 45, a 42% reduction compared to DCP. Due to more stable polarization rotation paths during AC poling, the domain structures in [011]- and [111]-oriented crystals remained relatively stable throughout the AC poling process. The results show that for different materials or even for different orientations of the same material, ACP will exhibit different effects, and in certain cases, it can lead to a decline in performance.

For third-generation Mn-doped PIN-PMN-PT single crystals, ACP has also been proven effective for performance optimization. To date, approximately 10 studies have reported the optimization of Mn-doped PIN-PMN-PT single crystals using the ACP method, as summarized in Table S3. The relative increase in $\varepsilon_{33}^{T}$/*ε*_0_ ranges from 13% to 84%, while the increase in *d*_33_ ranges from 15% to 61%; additionally, the mechanical quality factor *Q*_m_ can be improved by 17% to 118%. Figure 1c presents the best currently reported cases of ACP-optimized Mn-doped PIN-PMN-PT single crystals, as reported by Xu et al. [41]. In this study, applying the ACP method to Mn-doped PIN-PMN-0.32PT yielded a *d*_33_ of 2280 pC/N, a $\varepsilon_{33}^{T}$/*ε*_0_ of 6150, and a piezoelectric voltage constant (*g*_33_) of 41.8 × 10^−3^ Vm/N under a poling condition of 10.0 kV_pk_/cm at 1 Hz for 10 cycles. These values are significantly higher (60% for *d*_33_, 84% for $\varepsilon_{33}^{T}$/*ε*_0_) than those of DCP-processed single crystals, while maintaining a *Q*_m_ of 700.

In 2023, Tian et al. employed the ACP method to simultaneously improve *d*_33_ and *Q*_m_, while clarifying the underlying mechanism. Under a poling condition of 15.0 kV_pk_/cm at 25 Hz for 10 cycles, *d*_33_ increases by 16% from 1015 to 1174, and *Q*_m_ increases by 51% from 153 to 231. They proposed that, under an AC electric field, defect dipoles dissociate into isolated charged defects that accumulate at domain walls, strongly pinning domain wall motion while promoting ferroelectric dipole dynamics [42]. In 2024, Wu et al. utilized a combined DCP+ACP method to enhance *d*_33_ and *Q*_m_ in MPB-composition Mn-doped PIN-PMN-PT single crystals. This investigation achieved an 53% improvement in *d*_33_ (from 1435 pC/N to 2194 pC/N) and a 118% improvement in *Q*_m_ (from 193 to 420) compared to those obtained by DCP. The above studies indicate that the ACP method is also a promising tool for optimizing high-power piezoelectric materials and devices [43].

Rare-earth element doping is a well-established strategy to enhance the dielectric and piezoelectric properties of Pb-based relaxor ferroelectric single crystals and ceramics [87]. This improvement is attributed to the introduction of local structural heterogeneity and further flattening of the energy landscape in relaxor ferroelectrics induced by rare-earth doping. Recent studies indicate that the ACP method is also effective for optimizing the performance of rare-earth-doped relaxor ferroelectric single crystals, achieving remarkable ultra-high piezoelectric responses. In 2023, Yamashita et al. reported a piezoelectric constant of *d*_33_ = 4800 pC/N in ACP-processed La_2_O_3_-doped 0.70Pb(Mg_1/3_Nb_2/3_)O_3_-0.30Pb(Zr,Ti)O_3_ (PMN-PZT) single crystals with [001]_C_ orientation, grown using the solid-state single-crystal growth (SSCG) method. The optimal ACP condition was found to be 6.0 kV_rms_/cm triangular waves at 1 Hz for 10 cycles. Under these conditions, the *d*_33_ increases by approximately 71% from 2800 pC/N to 4800 pC/N, while $\varepsilon_{33}^{T}$/*ε*_0_ increases by approximately 42% from 10,000 to 14,200, and *k*_t_ exhibits an increase from 47.0% to 48.2%. To date, the *d*_33_ of 4800 pC/N in La-doped PMN-PZT single crystals processed by ACP represents the highest value reported for both DCP and ACP ferroelectric single crystals and ceramics without additional physical field assistance [88]. To preserve scientific rigor, two recent ultra-high piezoelectricity studies reported as preprints—which developed mechanically assisted poled PMN-33PT single crystals (*d*_33_ ~ 5000 pC/N) and continuous field-maintained PZT ceramics (*d*_33_ ~ 6850 pC/N) [89]—have been excluded from our primary comparison tables.

In 2024, Chen et al. investigated the effect of different ACP waveforms (sine, square, triangular, and sawtooth waves) on the performance optimization of Sm-doped 0.70PMN-0.30PT single crystals. Their results demonstrated that the optimal ACP condition for Sm-doped 0.70PMN-0.30PT single crystals is 7.59 kVrms/cm square waves at 0.5 Hz for 35 cycles. Under these optimal poling conditions, outstanding *d*_33_ and $\varepsilon_{33}^{T}$/*ε*_0_ values of 4520 pC/N and 13,290 were obtained, which are approximately 25% and 30% higher than those of the DCP counterpart, as illustrated in Figure 1c [90]. These remarkable properties exhibited by ACP-processed rare-earth-doped relaxor-based ferroelectric single crystals hold significant promise for the development of various other piezoelectric transducers.

In addition, the application of ACP for performance optimization has also been reported in other relaxor single crystal systems in recent years, as summarized in Table S4, including PMN-PYbN-PT, PIN-PT, and PZN-PT single crystals. In 2019, He et al. investigated the effects of ACP optimization on [001]_C_-oriented 0.52PMN-0.15PYbN-0.33PT single crystals. The results indicated that the $\varepsilon_{33}^{T}$/*ε*_0_ and *d*_33_ were 6800 and 2490 pC/N, respectively, for ACP-processed samples, which were 31% and 41% higher than those of the DCP counterpart; meanwhile, the ACP sample showed a 31% reduction in dielectric loss [44]. In 2021 and 2022, Xiong et al. ACP-induced performance optimization in [001]_C_-oriented 0.66PIN-0.34PT, 0.65PIN-0.35PT, and 0.655PIN-0.345PT single crystals. For 0.655PIN-0.345PT single crystals, ACP induced a 24.24% increase in $\varepsilon_{33}^{T}$/*ε*_0_ (from 2970 to 3690) and a 23.74% enhancement in *d*_33_ (from 1390 pC/N to 1720 pC/N) [45,46]. In 2024, Yamashita et al. investigated the effectiveness of ACP on [001]_C_-oriented 0.945PZN-0.055PT single crystals. ACP treatment achieved a $\varepsilon_{33}^{T}$/*ε*_0_ of 6680, *d*_33_ of 2760 pC/N, *g*_33_ of 46.7 × 10^−3^ Vm/N, and a *d*_33_ × *g*_33_ of 129 × 10^−12^ m^2^/N. These values were 19%, 31%, 10%, and 45% higher than those of DCP-processed PZN-PT single crystals, respectively [20]. The studies mentioned above demonstrate that the ACP method is capable of optimizing almost all currently known Pb-based relaxor ferroelectric single crystals. This fully demonstrates the universality and importance of ACP in optimizing the properties of relaxor ferroelectrics.

### 2.2. ACP Performance Optimization of Piezoelectric Ceramics

**Figure 2:**
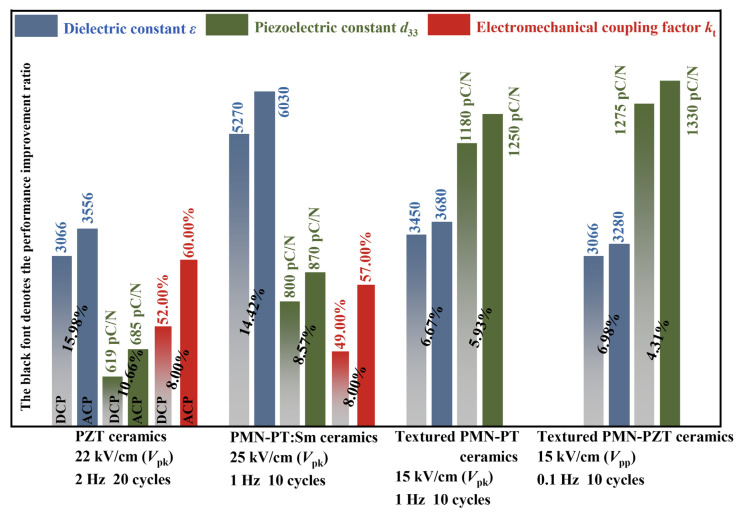
Comparison of dielectric constant *ε*, piezoelectric constant *d*_33_, and electromechanical coupling factor *k*_t_ between DCP and ACP for representative lead-based piezoelectric ceramics under optimal poling conditions.

Research on ACP-optimized relaxor ferroelectric single crystals has advanced considerably, leading to a gradual expansion of relevant studies on ACP-optimized ferroelectric ceramics. Despite approximately 20 studies addressing lead-based and lead-free ferroelectric ceramics processed by the ACP method, effective optimization remains largely limited to lead-based systems, as summarized in Figure 2.

In 2017, Tao et al. first attempted to apply ACP to ferroelectric ceramics, including PZT-5H (PZT), Ba_0.85_Ca_0.15_Ti_0.90_Zr_0.10_O_3_ (BCTZ), Bi_0.5_Na_0.5_TiO_3_-BaTiO_3_ (BNT-BT), and K_0.45_Na_0.55_Nb_0.96_Sb_0.04_O_3_-(Bi_0.75_Ho_0.25_)_0.5_Na_0.5_HfO_3_ (KNN-BHNH). Although this study proposes the concept of ACP-poled ferroelectric ceramics and demonstrates that the ACP process could yield piezoelectric properties and temperature stability comparable to the DCP process, the investigated ceramic systems did not exhibit significant performance improvements due to a lack of systematic exploration of poling conditions [91]. In 2021, Ma et al. achieved a significant enhancement in dielectric and piezoelectric properties of PZT-5H ceramics using the ACP method. Under poling conditions of 22 kV_pk_/cm at 2 Hz for 20 cycles, the $\varepsilon_{33}^{T}$/*ε*_0_, *d*_33_, and *k*_t_ increased by approximately 16% (from 3066 to 3556), 11% (from 619 pC/N to 685 pC/N), and 8% (from 52% to 60%), respectively. Domain structure analysis reveals that the ACP-induced improvement in the performance of PZT ceramics originates from the smaller domain size and higher domain wall density of the ACP-processed samples. This work represents the first study to evaluate the effectiveness of the ACP method for optimizing ferroelectric ceramic performance [92]. Subsequently, also in 2021, Yang et al. utilized the ACP method to improve the performance of [001]_C_ textured PMN-PT ceramics. Under poling conditions of 15 kV_pk_/cm at 1 Hz for 10 cycles, the $\varepsilon_{33}^{T}$/*ε*_0_ and *d*_33_ increased by approximately 6.67% (from 3450 to 3680) and 5.93% (from 1180 pC/N to 1250 pC/N), respectively. These findings indicate that the ACP method is not only effective for randomly oriented ferroelectric ceramics but also applicable to textured ceramics [93].

In 2025, Peng et al. applied the ACP method to Sm-doped PMN-PT ceramics. Under poling conditions of 25 kV_pk_/cm at 1 Hz for 10 cycles, the $\varepsilon_{33}^{T}$/*ε*_0_, *d*_33_, and *k*_t_ of ceramics increased by approximately 14% (from 5270 to 6030), 9% (from 800 pC/N to 870 pC/N), and 8% (from 49% to 57%), respectively [94]. In the same year, Tang et al. utilized the ACP to improve the properties of [001]_C_ textured PMN-PZT ceramics. At 15 kV_pp_/cm and 0.1 Hz for 10 cycles, the $\varepsilon_{33}^{T}$/*ε*_0_ and *d*_33_ exhibited an increase of approximately 7% (from 3066 to 3280) and 4% (from 1275 pC/N to 1330 pC/N), respectively [95]. Microscopic domain morphology analysis in this work indicates that grain boundaries restrict ferroelectric domain switching, thereby inhibiting domain growth across these boundaries. This phenomenon may account for the differences in performance optimization between ferroelectric single crystals and ceramics. Furthermore, between 2019 and 2025, several studies on ACP-optimized lead-free ferroelectric ceramics were reported, covering systems such as KNN-based, (Ba,Ca)(Ti,Zr)O_3_ (BCTZ), BiFeO_3_-BaTiO_3_ (BF-BT), and (Na,Bi)TiO_3_-BaTiO_3_ (NBT-BT) systems. In 2023, Wu et al. increased the *k*_t_ of Mn-doped 0.94NBT-0.06BT ceramics from 58.1% (DCP) to 74.7% using the ACP method while maintaining a nearly constant *d*_33_ of approximately 155 pC/N [96]. In 2025, Liu et al. applied the ACP to BCTZ ceramics. For the composition of Ba_0.88_Ca_0.12_Ti_0.89_Zr_0.11_O_3_, ACP led to excellent piezoelectric properties with *d*_33_ = 405 pC/N and *S*_max_/*E*_max_ = 620 pm/V, representing increases of 16% and 18%, respectively, compared to the DCP counterpart [97]. Also in 2025, Fu et al. achieved an improved balance between *d*_33_ and depolarization temperature (*T*_d_) in 0.92BNT-0.08BT ceramics using the ACP method. In this study, ACP significantly enhanced *d*_33_ from 65 pC/N to 161 pC/N while preserving a high *T*_d_ of 200 °C [98].

Despite these localized successes, lead-free piezoelectric ceramics present substantial challenges and inherent limitations when subjected to ACP. First, the optimization efficiency in lead-free systems remains lower than that of lead-based relaxor single crystals; for instance, many KNN-based ceramics poled by AC fields show negligible piezoelectric constant (*d*_33_) improvements, frequently remaining below 135 pC/N under standard conditions. Second, lead-free ceramics, particularly bismuth sodium titanate (NBT) systems, possess exceptionally high coercive fields (*E*_c_ > 30 kV/cm) and high electrical conductivity at room temperature. Applying high-amplitude AC electric fields at low frequencies to overcome these large energy barriers carries a severe risk of dielectric breakdown, requiring elevated processing temperatures or highly complex asymmetric waveforms that hinder scalable industrial manufacturing. Furthermore, the strong pinning effects of native defect dipoles (e.g., oxygen vacancies) in lead-free lattices restrict domain wall dynamics, making it difficult for the domains to follow the alternating field without causing significant self-heating and premature depolarization.

The above research indicates that the ACP method is a viable strategy for optimizing the dielectric and piezoelectric properties of ferroelectric ceramics. Although the optimization efficiency is significantly lower than that of relaxor-PT single crystals, the beneficial effects of ACP on ferroelectric ceramics remain evident. Given the widespread application potential and substantial market demand for ferroelectric ceramics, continued research into optimizing the electromechanical properties of ferroelectric ceramics using ACP is necessary. Furthermore, a noteworthy phenomenon is that ACP elevates the thickness electromechanical coupling factor (*k*_t_) of NBT-6BT ceramics by 28.6% (from 58.1% to 74.7%), as shown in Figure 2. For ultrasonic imaging transducers, it is well known that the *k*_t_ of the thickness vibration mode is the most crucial material parameter, as it directly determines the bandwidth and sensitivity of the device. Therefore, the significant improvement in *k*_t_ induced by ACP is of great significance for advancing the application of ferroelectric ceramics in high-end medical imaging ultrasonic transducers.

### 2.3. ACP Performance Optimization of Piezoelectric Composites

**Figure 3:**
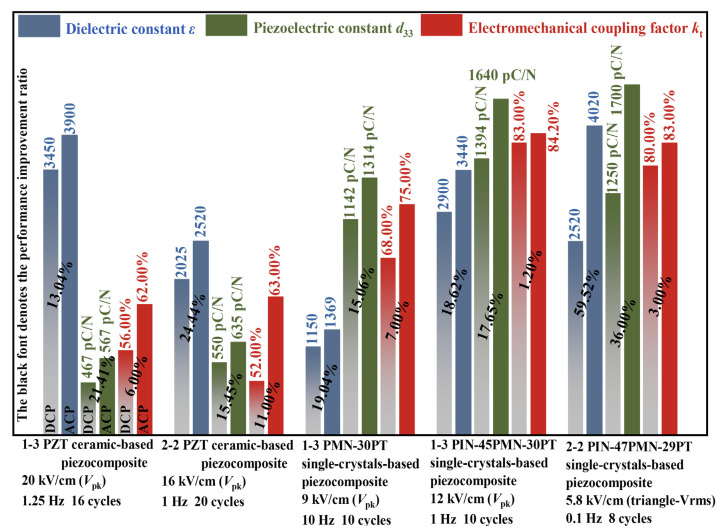
Comparison of dielectric constant *ε*, piezoelectric constant *d*_33_, and electromechanical coupling factor *k*_t_ for representative 1-3 and 2-2 piezocomposites subjected to DCP and ACP under optimal poling conditions.

In the field of ultrasonic transducers, particularly for medical imaging applications, the active layers typically consist of piezoelectric composites (piezocomposites) rather than bulk piezoelectric single crystals or ceramics. These composites are derived from the further processing of such bulk materials. By integrating an active piezoelectric phase with a passive polymer matrix, these composites offer significant advantages over conventional piezoelectric single crystals and ceramics, such as reduced acoustic impedance, high electromechanical coupling, and mechanical flexibility. Typically, 1-3 and 2-2 piezocomposites are widely used in single-element and array transducers, respectively. Following the continued progress in optimizing piezoelectric single crystals and ceramics using the ACP method, several studies have shown that although ACP increases *d*_33_ of the piezoelectric material, its *k*_t_ does not show a significant improvement. This is because ACP also increases the dielectric constant. More energy is dissipated or stored in forms such as dielectric loss rather than being fully converted into mechanical energy, resulting in *k*_t_ remaining unchanged or even decreasing. Since the electromechanical response of piezocomposites originates from the embedded piezoelectric phase, the demonstrated ability of ACP to enhance the properties of single crystals and ceramics suggests that it is also capable of optimizing the electromechanical performance of the corresponding piezocomposites. By cutting the blocky crystals into 1-3 or 2-2 piezoelectric composite structures, the dielectric constant can be reduced while enabling the piezoelectric phase to operate in a vibration mode similar to longitudinal expansion and contraction, thereby enhancing the *k*_t_ of the active layer. To date, research on ACP-optimized piezocomposites has primarily focused on 1-3 and 2-2 types based on piezoelectric single crystals and ceramics, materials that have been proven to be susceptible to ACP optimization for electromechanical properties, as summarized in Figure 3.

After observing the ACP optimization phenomenon in PZT ceramics, Ma et al. applied the ACP method to enhance the electromechanical properties of PZT-5H ceramic-based 1-3 piezocomposites in 2021 [99]. Under poling conditions of 20 kV_pk_/cm at 1.25 Hz for 16 cycles, $\varepsilon_{33}^{T}$/*ε*_0_, *d*_33_, and *k*_t_ of the PZT-ceramic-based 1-3 piezocomposites increased by approximately 13% (from 3450 to 3900), 21% (467 pC/N to 567 pC/N), and 6% (from 56% to 62%), respectively, compared to those of the DCP counterparts. Consistent with the microstructural mechanisms in bulk PZT ceramics, domain pattern analysis reveals that the performance improvement in these composites stems from the distinct, regularly striped domain structure, the increase in the domain wall density and the generation of a regular striped pattern, elevating *d*_33_ of PZT composites by 21.4%.

This study demonstrates, for the first time, the optimization effect of ACP on ceramic-based piezocomposites and indicates that ACP-poled samples are promising candidates for ultrasonic transducers. Later, in 2023, Ma et al. further verified the effectiveness of ACP optimization in PZT-5H ceramic-based 2-2 piezocomposites [92]. Under poling conditions of 16 kV_pk_/cm at 1 Hz for 20 cycles, $\varepsilon_{33}^{T}$/*ε*_0_, *d*_33_, and *k*_t_ of 2-2 piezocomposites increased by approximately 24% (from 2025 to 2520), 15% (550 pC/N to 635 pC/N), and 11% (from 52% to 63%), respectively, relative to the DCP counterparts.

Recent studies have also reported ACP optimization in piezocomposites based on Pb-based relaxor ferroelectric single crystals. In 2022, Ma et al. optimized 1-3 piezocomposites based on [001]_C_-oriented 0.70PMN-0.30PT single crystals using the ACP method under poling conditions of 9 kV_pk_/cm at 10 Hz for 10 cycles. Under these conditions, the $\varepsilon_{33}^{T}$/*ε*_0_, *d*_33_, and *k*_t_ of the single-crystal-based 1-3 piezocomposites increased by approximately 19% (from 1150 to 1369), 15% (1142 pC/N to 1314 pC/N), and 7% (from 68% to 75%), respectively, compared to DCP counterparts [100]. In 2023, Jia et al. applied the ACP method to enhance the dielectric and piezoelectric properties of 1-3 piezocomposites based on 0.25PIN-0.45PMN-0.30PT single crystals. Under poling conditions of 12 kV_pk_/cm at 1 Hz for 10 cycles, compared with the DCP method, $\varepsilon_{33}^{T}$/*ε*_0_ increased by 19% from 2900 to 3440, *d*_33_ was enhanced by 18% from 1394 pC/N to 1640 pC/N, and *k*_t_ increased from 83% to 84.2%. This work verified the effectiveness of ACP optimization for PIN-PMN-PT single-crystal-based piezocomposites [101]. Also in 2023, Ma et al. further optimized the performance of PIN-PMN-PT single-crystal-based 2-2 piezocomposites. Under the poling condition of 5.8 kV_rms_/cm triangle waves at 0.1 Hz for 8 cycles, the $\varepsilon_{33}^{T}$/*ε*_0_, *d*_33_, and *k*_t_ of [001]_C_-oriented 0.24PIN-0.47PMN-0.29PT 2-2 piezocomposites increased by approximately 60% (from 2520 to 4020), 36% (from 1250 pC/N to 1700 pC/N), and 3% (from 80% to 83%), respectively [102].

In addition, in 2024, Ning et al. investigated the thickness and temperature-dependent properties of [001]_C_-oriented PIN-PMN-PT single-crystal-based 1-3 piezocomposites subjected to the ACP and DCP methods. Their results demonstrate that the dielectric and electromechanical properties are effectively enhanced by ACP, and a scaling effect was observed in samples thinner than 400 μm. Additionally, low-thickness piezocomposites exhibited inferior $\varepsilon_{33}^{T}$/*ε*_0_, *d*_33_, and *k*_t_, along with reduced performance improvements under ACP. Regarding temperature stability, all properties improved as temperature increased, a trend that was independent of poling conditions. This study offers valuable insights for selecting appropriate poling methods, thereby facilitating the design and fabrication of transducers [103].

The aforementioned studies demonstrate that ACP is an effective method for optimizing single-crystal-based and ceramic-based piezocomposites. The efficient optimization of 1-3 and 2-2 piezocomposites using the ACP method establishes a critical material foundation for advancing high-performance single-element and phased array ultrasonic transducers. Furthermore, a comparison reveals valuable information: ACP enhances the *k*_t_ of piezocomposites to a greater extent than that of single crystals. Since increasing the *k*_t_ of piezoelectric materials concurrently improves transducer bandwidth and sensitivity, achieving a high *k*_t_ is essential for fabricating high-performance medical imaging transducers.

### 2.4. Optimization Mechanism of ACP on Piezoelectric Materials

The performance enhancement of piezoelectric materials by the ACP method primarily arises from the unique microstructural evolution induced by the AC electric field. During ACP, polarization periodically reverses due to the alternating field, driving continuous ferroelectric domain reconfiguration. The resulting domain structure, which directly determines piezoelectric properties, is influenced by parameters such as AC amplitude, frequency, and poling cycles.

**Figure 4:**
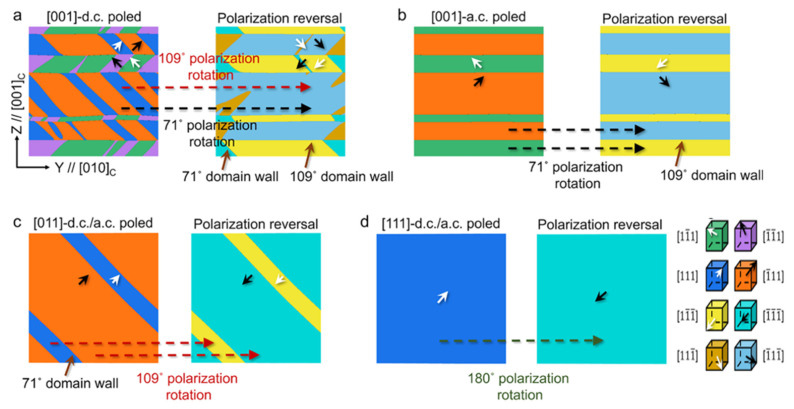
Phenomenological illustration of domain structural evolution for various oriented R crystals under ACP and DCP: (**a**) [001]-d.c. poled, (**b**) [001]-a.c. poled, (**c**) [011]-d.c. and a.c. poled, (**d**) [111]-d.c. and a.c. poled samples and respective polarization reversal process. The black and red dashed arrow in (**a**,**b**) give the corresponding 71° and 109° polarization rotation regions, respectively. The different colors represent ferroelectric domains with polarization along different directions, and detailed polarization direction is depicted in the upper right corner of the figure. The arrows denote polarization directions, and the color of arrow corresponds to the positive (white) and negative (black) polarization components along [100] direction [29].

Thermodynamically, domain switching initiates under an external electric field when *E*_i_Δ*P*_i_ ≥ 2*P*_s_*E*_c_, where *E*_i_ is the electric field amplitude, Δ*P*_i_ signifies the polarization change, *P*_S_ corresponds to the spontaneous polarization, and *E*_C_ denotes the coercive fields [104,105]. This criterion suggests the need for sufficient electric field strength to overcome the energy barrier for domain switching. In ACP (and DCP), low frequencies promote domain coalescence into larger structures, whereas high frequencies suppress lateral domain growth, leading to frequency-dependent domain width variations. The number of poling cycles plays another critical role, as increasing cycles within reasonable limits induces merging of adjacent 71° domains and expansion of 109° domains, yielding a uniform, simplified domain structure in [001]_C_-oriented rhombohedral/MPB relaxor ferroelectric single crystals, as shown in Figure 4. This effect is attributed to prolonged AC cycling modulating Landau free energy through domain evolution, optimizing stability, refining domains, and enhancing electromechanical properties.

Although ACP has been validated mainly in relaxor single crystals, the underlying mechanisms that govern domain evolution remain debated, with the impact on domain size being a central point of contention. Experimental studies have reported both an increase and a decrease in domain size following ACP, yet both outcomes have been correlated with enhanced electromechanical properties. Rather than viewing these observations as contradictory, we here propose a unified framework that reconciles them by considering the initial domain configuration and the applied ACP parameters. The overall effect of ACP can be understood as driving the ferroelectric domain system toward a more energetically stable and dynamically responsive state, irrespective of whether the absolute domain width increases or decreases. The resulting property enhancement arises from the optimization of two interdependent factors: (i) the intrinsic piezoelectric response, which is closely tied to the uniformity of the domain pattern and the release of internal lattice strain, and (ii) the extrinsic response, which is governed by domain wall mobility and density.

**Figure 5:**
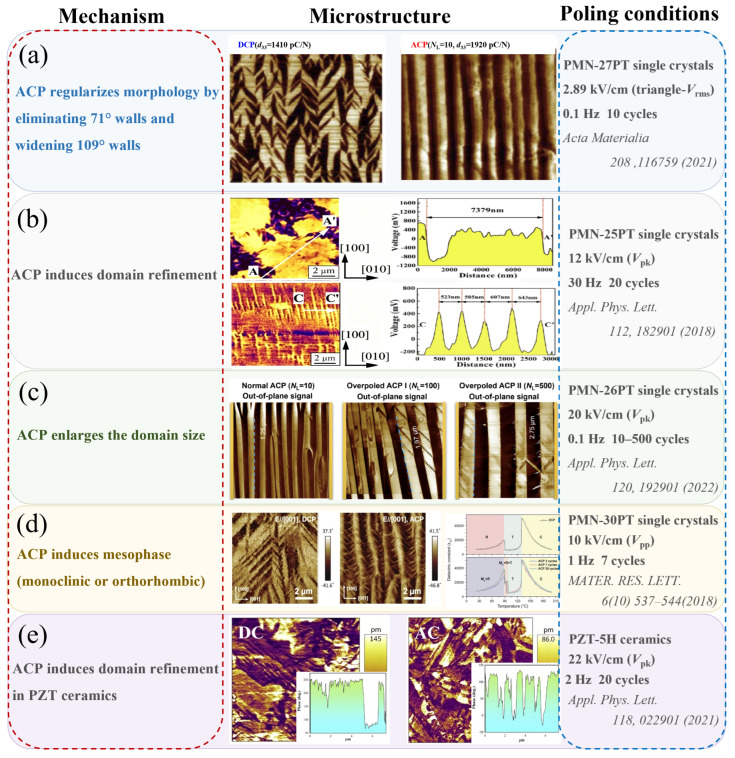
Evolution of domain structures under several typical ACP conditions: (**a**) Domain morphology of [001]_C_-oriented 0.73PMN-0.27PT single crystals under DCP and ACP [66]; (**b**) Domain morphology in [001]_C_-oriented 0.75PMN-0.25PT single crystals under DCP and high-frequency ACP [13]; (**c**) Evolution of the domain structure in [001]_C_-oriented 0.74PMN-0.26PT single crystals as a function of poling cycles under low-frequency ACP conditions [32]; (**d**) Domain morphology in [001]_C_-oriented 0.70PMN-0.30PT single crystals under DCP and ACP [67]; (**e**) Domain morphology in PZT ceramics under DCP and ACP [52].

The variation in domain size evolution during the ACP process may stem from differences in poling conditions. Specifically, higher poling frequencies tend to induce smaller domains, whereas lower frequencies favor the formation of larger domains. Furthermore, combining low frequencies with an increased number of poling cycles can promote domain growth. Figure 4 illustrates the evolution of domain structures under several typical ACP conditions reported in the literature. Figure 5a compares the domain morphology of [001]_C_-oriented 0.73PMN-0.27PT single crystals under DCP and ACP. The ACP was conducted using a 0.1 Hz triangular wave at 2.89 kVrms/cm for 10 cycles. It is observed that ACP induces a uniform and simplified domain pattern characterized by the elimination of 71° domain walls and the widening of 109° domains. Consequently, the *d*_33_ significantly improved from 1410 pC/N to 1920 pC/N following the regularization of the domain structure by ACP. Figure 5b presents a comparison of domain morphology in [001]_C_-oriented 0.75PMN-0.25PT single crystals under DCP and high-frequency ACP (12 kV_pk_/cm, 30 Hz, 20 cycles). The high-frequency poling condition induced a refined domain structure with smaller dimensions in the single crystals. This structural refinement also contributed to a significant enhancement in the electromechanical performance of the PMN-PT single crystals. Figure 5c displays the evolution of the domain structure in [001]_C_-oriented 0.74PMN-0.26PT single crystals as a function of poling cycles under low-frequency ACP conditions (20 kV_pk_/cm, 0.1 Hz, 10–500 cycles). The size of the striped domains was observed to increase continuously as the number of poling cycles rose from 10 to 500. This indicates that, under low-frequency ACP, increasing the number of poling cycles can regulate domain size within a specific range. However, the optimal performance in this case was achieved with only 10 cycles (*d*_33_ = 1660 pC/N). This excessive domain size resulting from an increased number of poling cycles represents an overpoling effect that fails to yield optimal performance. Therefore, ACP proves to be an effective technique for controlling domain structures. The primary focus of ACP research lies in designing and constructing domain structures that achieve optimal or customized material properties through appropriate poling conditions [13,34,47,48,97].

It has also been reported that ACP can induce metastable phases, such as monoclinic and orthorhombic phases, in relaxor single crystals, as shown in Figure 5d. When the exchange polarization introduces the metastable phase, it will cause a ferroelectric-to-ferroelectric phase transition in the low-temperature region. This will lead to a decrease in the temperature stability of dielectric and piezoelectric properties, and when the temperature crosses the ferroelectric-to-ferroelectric phase transition, the sample will become depolarized. Therefore, although the introduction of the metastable phase through exchange polarization has the potential to enhance the performance of the device, it also has the disadvantage of reducing the stability of the device performance. However, this phenomenon exhibits a certain degree of material specificity and is not a universal occurrence across all materials. This phenomenon may be attributed to variations in material composition, growth method, and intrinsic microstructures. Figure 5e gives a comparison of domain morphology in PZT ceramics under DCP and ACP. Similar to the majority of current reports on ACP optimization of ceramic properties, ACP enhances electromechanical performance in PZT ceramics by reducing domain size. Furthermore, a study by Tang et al. on the ACP optimization of textured PMN-PZT ceramics indicated that grain orientation and grain boundaries significantly influence the optimized properties [55]. It suggests that, due to the influence of grain boundaries, ceramics differ significantly from single crystals, as domain switching and growth are constrained by orientation variations between grains. Phase-field simulations further reveal that grain boundaries impede domain growth in polycrystals, thereby hindering the formation of larger domains. These results provide important insights into the physical mechanisms explaining why the ACP optimization efficiency for ceramics is lower than that for single crystals. The mechanism by which ACP optimizes piezocomposite materials is primarily attributed to the optimization mechanism of ACP for the corresponding piezoelectric framework materials.

Herein, we highlight several critical issues and ongoing challenges that warrant focus for the further advancement and objective evaluation of the ACP method:

1. Delving deeper into the physical mechanisms and reproducibility of the ACP method. The influence of domain structure on the piezoelectric response remains a complex and highly sensitive phenomenon. Although ACP technology offers a powerful tool for tuning microscopic domain structures (e.g., inducing smaller domain sizes and higher domain wall densities to enhance macroscopic properties), the optimal AC electric field parameters—such as waveform, frequency, and temperature—often exhibit significant variability across different material compositions. Addressing the challenges of process reproducibility and understanding the exact boundary conditions under which ACP becomes less effective are essential for guiding the deterministic design of high-performance materials.
2. Balancing and optimizing a broader range of electromechanical performance parameters for practical device applications. Current ACPd research predominantly focuses on boosting the dielectric constant, piezoelectric coefficient *d*_33_, and longitudinal electromechanical coupling factor *k*_33_. However, practical device design demands a balanced combination of “soft” and “hard” piezoelectric characteristics. For certain configurations, the improvement of specific parameters (such as the thickness electromechanical coupling factor *k*_t_) remains limited through conventional ACP. Future research must explore the limits of ACP or combine it with other modification methods to simultaneously enhance the off-resonance figure of merit (FOM) defined by (*d*_33_ × *g*_33_)/tan *δ* and the on-resonance FOM defined by *Q*_m_ × *k*^2^, thereby mitigating the trade-off between loss and response.
3. Expediting the broader application of the ACP method to a wider range of material systems, particularly polycrystalline ceramics. At present, systematic ACP investigations are heavily concentrated on relaxor-based single crystals, while investigations into the ACP effects on polycrystalline ceramics remain limited. Considering the dominant role of piezoelectric ceramics in electromechanical device applications, it becomes imperative to implement and extensively promote ACP technology within ceramic research.

## 3. Performance Relationship Between Piezoelectric Materials and Ultrasound Transducer

### 3.1. Performance Parameters of Piezoelectric Materials

Electromechanical coupling coefficient: Represented by *k*, this coefficient serves as a fundamental parameter indicating the efficiency of energy conversion between electrical and mechanical forms in piezoelectric materials. It quantifies the degree of interaction between the electrical and mechanical responses. Mathematically, *k* is defined as: *k*^2^ = (Mechanical output)/(Electrical input) or *k*^2^ = (electrical output)/(mechanical input). The *k* is a dimensionless parameter, and its value is closely associated with the degree of poling.

Piezoelectric strain constant: The piezoelectric strain constant, denoted as *d* (*d* = ∂*S*/∂*E*), serves as a key metric for quantifying the material response along a specific direction. It characterizes the magnitude of electric charge generated per unit of applied mechanical stress, as well as the mechanical strain or deformation resulting from a unit of applied electric field. Mathematically, this relationship can be expressed as: *d* = *Q*/*F*, where *Q* represents the electric charge accumulated on the sample surface, while *F* stands for the force exerted on the sample.

Piezoelectric voltage constant: The piezoelectric voltage constant (*g* = ∂*E*/∂*T*), commonly referred to as the open-circuit coefficient, serves as a metric for assessing the capacity of piezoelectric materials to produce a significant voltage in response to an applied unit stress. The *g* constant is mathematically related to the *d* constant by the equation: *g* = *d*/*ε*, where *ε* represents the absolute permittivity, which is the product of the relative dielectric constant (*ε*_r_) and the permittivity of free space (*ε*_0_).

Dielectric constant: The dielectric constant (*ε*) characterizes the extent to which the electric field inside a material is reduced relative to that in a vacuum. It assesses the ability to be polarized by an external electric field, which results in the separation of internal charges. Materials with a higher *ε* are capable of storing more electric charge under an applied field, thereby exhibiting greater effectiveness in polarizing and redistributing charges.

Mechanical quality factor: The mechanical quality factor, denoted as *Q*_m_, evaluates the magnitude of energy dissipation or loss during mechanical vibration. A high *Q*_m_ value signifies minimal energy loss and superior efficiency for converting mechanical vibrations to electrical signals, or vice versa, particularly at a specific resonant frequency. The *Q*_m_ serves as an indicator of the sharpness or narrowness of the mechanical resonance peak. Mathematically, *Q*_m_ is defined as: *Q*_m_ = (resonant frequency)/(FWHM), where the resonant frequency refers to the frequency at which the material exhibits its maximum response to mechanical vibrations, while FWHM represents the full width at half maximum.

Dielectric loss: The dielectric loss factor, denoted as tan *δ*, characterizes the energy dissipation within a dielectric material when subjected to an alternating electric field. It quantifies the ratio of energy lost as heat to the energy stored by the material during one full cycle of the electric field. tan *δ* is particularly critical for applications involving alternating fields. Mathematically, tan *δ* is expressed as: tan *δ* = (imaginary part of complex permittivity)/(real part of complex permittivity).

Frequency constant: The frequency constant (*N*) is a fundamental material parameter that characterizes the relationship between the resonant frequency and the physical dimensions of a piezoelectric resonator. The *N* is an intrinsic property that remains largely independent of the specific geometry of the sample for a given vibrational mode. To maintain resonance at a specific harmonic, the product of the resonant frequency (*f*) and the corresponding linear dimension controlling the sound wave propagation must remain constant. Mathematically, the frequency constant is defined as: *N* = *f* × *L*; where *f* is the resonant frequency, and *L* is the dimension of the ceramic resonator in the direction of vibration [106].

The high-density domain walls induced by ACP significantly improve both the dielectric constant and the piezoelectric strain coefficient. Consequently, the electromechanical coupling coefficient is moderately enhanced, whereas the piezoelectric voltage coefficient and frequency constant remain largely unaffected. However, the enhanced domain wall mobility introduces additional energy dissipation mechanisms, leading to a pronounced reduction in the mechanical quality factor and a concurrent increase in dielectric loss.

### 3.2. Piezoelectric Material Properties and Their Correspondence with Transducer Performance

**Figure 6:**
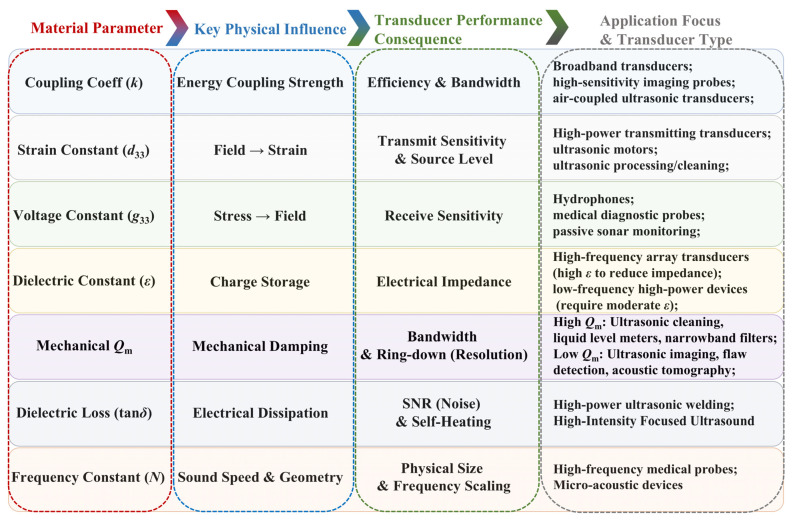
Mapping relationship between intrinsic piezoelectric material parameters and ultrasound transducer performance metrics.

The performance of an ultrasound transducer, during both transmission and reception, is determined by the intrinsic physical parameters of the piezoelectric material. These parameters govern electromechanical conversion efficiency, spectral response, and sensitivity limits. This section provides a comprehensive analysis of these key properties and their direct implications for transducer performance, as illustrated in Figure 6.

As mapped in Figure 6, these coupled material parameters dictate the performance across diverse clinical imaging modalities. For instance, high *d*_33_ and low dielectric loss are highly crucial for continuous-wave High-Intensity Focused Ultrasound (HIFU) to prevent premature thermal damage. In contrast, high-frequency Intravascular Ultrasound (IVUS) and high-resolution ophthalmic imaging require high clamped dielectric constants ($\varepsilon_{33}^{S}$) to match highly miniaturized aperture sizes, while shear wave elastography benefits from optimized electromechanical coupling factor *k_t_* to maximize localized acoustic radiation force impulses.

Electromechanical coupling coefficient (*k*)

This coefficient is central to transducer design as it defines the maximum theoretical efficiency for energy conversion between electrical and mechanical forms. It directly determines the usable bandwidth, which is approximately proportional to *k*. Consequently, high *k* values present broad bandwidths suitable for pulse-echo applications, whereas low values result in narrow bands. Furthermore, a higher *k* minimizes insertion loss, ensuring efficient signal power transfer through the system.

Piezoelectric strain constant (*d*_33_)

The *d*_33_ primarily dictates the source level. It establishes the magnitude of mechanical displacement or vibration velocity produced for a given driving voltage. Therefore, materials with higher *d*_33_ values generate greater acoustic pressure. This parameter is particularly critical for high-intensity applications requiring maximum displacement.

Piezoelectric voltage constant (*g*_33_)

In the receiving mode, *g*_33_ determines the open-circuit voltage generated in response to incident acoustic pressure. Higher *g*_33_ values produce a larger voltage output from weak signals. While this constant enhances signal amplitude, it works in conjunction with low dielectric loss to optimize the signal-to-noise ratio (SNR) by effectively suppressing noise.

Dielectric constant (*ε*)

The dielectric constant determines the clamped capacitance and further electrical impedance of the transducer. High dielectric constants lead to low electrical impedance, whereas low constants result in high impedance. Consequently, a careful selection of the dielectric constant is essential for the effective design of impedance-matching networks.

Mechanical quality factor (*Q*_m_)

The *Q*_m_ value is inversely proportional to bandwidth. High *Q*_m_ produces sharp, narrow resonant peaks and prolonged oscillation (ring-down), which degrades axial resolution in imaging. Conversely, low *Q*_m_ provides strong damping, shortens the pulse duration, and yields a broad bandwidth. Thus, while high *Q*_m_ is preferred for continuous wave applications, low *Q*_m_ is essential for high-resolution pulsed systems.

Dielectric loss (tan *δ*)

Dielectric loss significantly affects the signal-to-noise ratio (SNR) and thermal management. In the receiving mode, loss generates thermal noise. A higher loss increases thermal noise voltage, thereby degrading the SNR. Additionally, high loss causes substantial self-heating under high drive conditions. Excessive internal heating can raise the temperature above the Curie point or cause thermal aging, leading to permanent degradation of piezoelectric properties and limited power capacity.

Frequency Constant (N)

The frequency constant governs the dimensional scaling of the device, defining the physical thickness or length required for a specific operating frequency. For high-frequency transducers, materials with higher *N* values allow for thicker elements. This characteristic alleviates manufacturing difficulties compared to materials that require extremely thin wafers.

### 3.3. Performance Parameters of Ultrasound Imaging Transducers

Acting as the critical intermediary between electronic back-ends and biological subjects, ultrasound transducers perform the dual role of acoustic wave projectors and receivers. Upon receiving an electrical excitation, the piezoelectric components transform this energy into mechanical oscillations, thereby launching ultrasonic waves into the target anatomy. During the subsequent reception interval, the device captures the echoes reflected from internal tissue structures and reconverts the resulting acoustic pressure into electrical signals. Ultimately, imaging metrics, such as axial and lateral resolution alongside the signal-to-noise ratio, are governed by the physical construction of the transducer.

**Figure 7:**
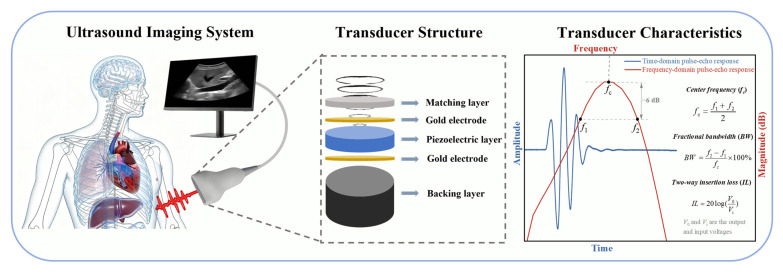
Schematic representation of ultrasound imaging system, multilayer transducer structure, and typical pulse-echo responses in time and frequency domains.

Figure 7 illustrates a schematic diagram of the ultrasound imaging system, the internal structure of the transducer, and the corresponding time-domain and frequency-domain signal representations. The transducer is composed of a matching layer, a piezoelectric layer, a backing layer, and metal electrodes. In these components, the piezoelectric layer is the most essential component. The performance of ultrasound imaging transducers is governed by several parameters. Center frequency determines the balance between resolution and penetration depth; while higher frequencies yield better resolution, they limit the depth of imaging. Bandwidth represents the range of frequency responses and defines axial resolution, where a wider bandwidth enables the production of short pulses to improve image detail. Insertion loss quantifies the signal energy attenuation during transmission, serving as an indicator of the efficiency of the internal electrical and acoustic interfaces. Sensitivity refers to the capability of the transducer to convert weak acoustic pressure into electrical signals. These two factors are inversely related: lower insertion loss corresponds to higher receiving sensitivity, a feature that is crucial for deep tissue imaging and ensuring a high signal-to-noise ratio.

The center frequency and −6 dB bandwidth are determined by the Fast Fourier Transform (FFT) spectrum of received transducer signals, as shown in Figure 7, using the following equations [107]:

(1) $$f_{c}=\frac{f_{1}+f_{2}}{2}$$

(2) $$BW=\frac{f_{2}-f_{1}}{f_{c}}\times 100\%$$

where *f*_1_ and *f*_2_ are the lower and upper −6 dB frequencies, respectively. The two-way insertion loss (relative pulse-echo sensitivity) is calculated using the following equation:

(3) $$IL=20\log(\frac{V_{0}}{V_{i}})$$

where *V*_o_ and *V*_i_ refer to the output and input voltages of the transducer, respectively.

For an air-backed piezoelectric transducer, the theoretical limit of the −6 dB relative bandwidth is related to the electromechanical coupling coefficient *k* of the piezoelectric material by:

(4) $$BW_{-6\mathrm{dB}}=\frac{4k^{2}}{\pi \sqrt{1-k^{2}}}$$

For instance, the 1-3 PZT-5H composite material prepared by Zhu et al. had *k*_t_ values of 0.66 and 0.75 under ACP and DCP treatments. The theoretical calculation showed that the bandwidth of ACP was 35% higher than that of DCP, which was similar to the 23% experimental result [108]. When *k*^2^ ≪ 1, the formula can be simplified into the approximation commonly used in engineering:

(5) $$BW_{-6\mathrm{dB}}\approx \frac{4k^{2}}{\pi }$$

Consequently, enhancing the electromechanical coupling coefficient *k* of piezoelectric materials is crucial for optimizing the bandwidth of transducers.

In an ultrasound imaging system, the ultrasound transducer must perform both transmitting and receiving functions. Consequently, the two-way insertion loss and sensitivity are determined by the efficiency of both processes. Transmit sensitivity, which converts electrical energy into acoustic energy, depends on the piezoelectric coefficient *d*. Receiving sensitivity, which converts acoustic energy back into electrical energy, depends on the coefficient of *g* = *d*/*ε*. Therefore, the two-way sensitivity is directly proportional to the figure of merit *d* × *g* = *d*^2^/*ε* [109]. Given the relationship *k*^2^ = *d*^2^/(*sε*), where *s* is the elastic compliance constant, it is evident that two-way sensitivity and insertion loss are also largely determined by *k*. This suggests that optimizing *k* is a primary objective when developing piezoelectric materials for ultrasound imaging transducers. Fortunately, ACP has proven effective in optimizing the electromechanical coupling ability of piezoelectric materials, particularly for piezocomposite materials. Furthermore, ACP shows potential for suppressing spurious-mode vibrations, which helps enhance the *k* of piezoelectric vibrators. These findings indicate that ACP is a promising method for optimizing the performance of ultrasound imaging transducers.

## 4. Performance Optimization of Ultrasound Imaging Transducer by ACP Method

In the first two parts, we respectively examined the significant enhancement of the intrinsic properties of piezoelectric materials achieved by the AC (Alternating Current) polarization method, as well as the quantitative relationship between the key parameters of piezoelectric materials and the performance of ultrasonic transducers. These relationships clearly indicate that the high dielectric constant, high electromechanical coupling coefficient, and moderate increase in dielectric constant achieved through AC polarization can directly translate into an improvement in the sensitivity of the transducer and an expansion of the pulse-echo bandwidth. However, transitioning from the optimized piezoelectric materials to high-performance ultrasonic imaging transducers is not a simple “mapping” of performance; it is a complex systems engineering problem involving multi-physics field coupling, micro-processing technology, and acoustic matching. The domain structure evolution, internal built-in electric field distribution changes, and material aging behavior caused by AC processing will generate new problems in the cutting of transducer elements, matching layers, and electrode fabrication, which are different from those of traditional DC polarization (DCP). At the same time, how to maximize the material gain brought by AC to the final device and meet the strict requirements of medical imaging in terms of array consistency, sound field control, and long-term reliability remains a core question that needs to be answered in this field. Therefore, this part focuses on the performance optimization of ultrasonic imaging transducers based on the AC polarization method, from a single array element to an array, from material gain to system synergy, and systematically reviews the important progress in recent years. Table S5 comprehensively summarize relevant reports on transducers under ACP and DCP from 2020 to 2025 [23,108,110,111,112,113,114,115,116,117,118,119].

At the single-element transducer level, the improvement effect of ACP on sensitivity and bandwidth has been widely verified. In 2020, Wan et al. conducted a transducer study on PMN-0.29PT, and both the simulation and experimental results showed that the transducers prepared from single crystals processed by ACP had their sensitivity and bandwidth enhanced. The experimental results indicated that compared with DCP, the sensitivity of the single-crystal transducers processed by ACP increased by 26%, and the bandwidth was widened by 4%, mainly due to the increase in *d*_33_, dielectric constant, and *k*_t_ of the processed single crystals [110]. Guan et al. increased *d*_33_ and *k*_t_ of PIN-PMN-PT single crystals by 37% and 3.5%, respectively, and integrated them into a 10 MHz surface imaging transducer, achieving an echo amplitude of 1.7 V, a −6 dB bandwidth of 55.04%, and a relative sensitivity of 35.34 dB, which were 0.54 V, 10.73%, and 3.3 dB higher than those of DCP crystal transducers, respectively. At the same time, compared with standard DCP-treated PZT ceramic transducers, the bandwidth was increased by 28.52%, and the sensitivity was increased by 5.58 dB [120]. The comparative study by Wu et al. further demonstrated that the 10 MHz transducer prepared using ACP single crystals not only exhibited better impedance and higher signal-to-noise ratio, but also showed more excellent durability in long-term high-input conditions, and the acoustic pressure output performance of the ACP transducer was superior in diagnostic and therapeutic dual-mode applications [111]. These works strongly prove that the material performance improvement brought by ACP can directly translate into significant improvements in the acoustic response of single-element transducers.

It is worth noting that the suppression effect of ACP on the transverse vibration modes provides a unique advantage for the design of high-performance array transducers. In 2020, Xu et al. found that compared with DCP polarization, the *k*_t_ value of the PMN-0.25PT single crystal after ACP treatment did not change much, but *k*_33_ and *k*_31_ were increased by 1.4% and 2.3%, respectively. The parameter *k*_15_ of the shear mode after ACP treatment decreased from 23.8% to 15% [121]. Meanwhile, based on these parameters, they conducted further simulation analysis on the piezoelectric oscillators approaching the longitudinal expansion vibration mode, and the results showed that under different aspect ratios, k_33_’ after ACP treatment was higher than that after DCP treatment, especially in the high aspect ratio case; the samples treated with DCP exhibited transverse resonance modes, while the samples treated with ACP only exhibited transverse resonance modes at higher aspect ratios. In the transducer simulation, the samples treated with ACP also demonstrated a suppression effect on transverse vibration, and this finding indicates that the ACP-treated piezoelectric materials have advantages in 1-3 composite piezoelectric transducers and phased array transducers, which is conducive to expanding the working bandwidth of high-density medical array elements. In the same year, Zhang et al. further explained through finite element modeling that the ACP method can improve the longitudinal transmission efficiency of acoustic energy, reduce the coupling of transverse vibration, and thereby reduce the interaction between adjacent units of the phased array, and increase the bandwidth of the transducer. Compared with DCP, the crosstalk between elements in the ACP array was reduced by 0.91 dB, and the bandwidth was increased by 7.2% [112].

In the field of composite material transducers, ACP also shows significant effects. Zhu et al. systematically compared the processing effects of ACP on 1-3 composite materials based on PZT ceramics and PMN-PT single crystals. The results showed that compared with DCP, the *d*_33_ of the two types of composite materials increased by 151 pC/N and 174 pC/N, respectively, after ACP treatment, and *k*_t_ increased by 9% and 15%, respectively. Correspondingly, the performance of the transducers prepared from 1-3 composite materials treated with ACP was also significantly improved. The −6 dB bandwidth of the transducers based on ceramic and single-crystal substrates after ACP treatment increased by 22.3% and 34.9%, respectively [108]. Hang et al. confirmed in the PMNT/epoxy resin 1-3 composite material that the regular strip domains and dense domain walls induced by ACP led to an increase of 2.31% in *k*_t_ compared with DCP, and the bandwidth of the transducer was accordingly increased by 6.27% [113]. Li et al. studied the effect of ACP in PIMNT/epoxy resin 2-2 composite transducers and achieved a 63.98% 6 dB bandwidth and a relative sensitivity of 27.78 dB, which were 6.67% and 2.42 dB higher than those processed with DCP, respectively [114]. These studies show that ACP has universal optimization effects on different composite configurations of piezoelectric composites.

There is a mutual constraint relationship between the bandwidth and sensitivity of ultrasonic transducers. Their performance not only depends on the inherent parameters of the material, but also relies on the collaborative design at the system level. Ma et al. applied ACP technology to PZT-5H linear array piezoelectric composite materials, achieving a sensitivity of up to 26 dB and a bandwidth increase of 14.26% compared to DCP. This improvement not only originated from the ACP’s improvement of *d*_33_ (increased by 15.45%) and *k*_t_ (increased by 21.15%) of the PZT-5H base composite material, but was the result of the collaborative optimization of the entire transducer system level, including the 24.44% increase in dielectric constant caused by ACP, which made impedance matching easier. Combined with appropriate matching layers and backfill layer design, the final achievement was a leap in device performance [118]. In 2022, Sun et al. developed large-sized PMN-0.3PT single-crystal plates for phased array transducers for cardiac detection at 40 °C. After cutting, the mechanical processing-induced performance decline was restored at the actuator level through DCP. This ACP-DCP process-processed single crystal oscillator had a *k*_33_′ that was approximately 4% higher than the traditional DCP-DCP process, providing a new path for the preparation of high-quality phased arrays [49]. Xu et al. further conducted experimental verification on a 64-element, 2.8 MHz phased array. The ACP-optimized PMN-PT array achieved a bandwidth increase of 7.90% and outperformed the DCP array in terms of longitudinal resolution and penetration depth [121]. Zhang et al. optimized the single-crystal performance through ACP and introduced a hard tungsten carbide acoustic reflection layer, achieving a bandwidth of 89.3% and a sensitivity of 8.39 dB for the phased array. Combining the optimization of phased array element performance with the design of the structural framework, it is expected to further enhance the resolution and penetration depth of ultrasonic imaging, addressing the existing problems in current flexible and stretchable ultrasonic devices due to insufficient bandwidth and poor sensitivity performance [115].

In terms of special applications and system integration, ACP also demonstrates unique value. Jiang et al. used the ACP process under medium-temperature conditions to increase PMN-0.27PT’s dielectric constant by 16%, *k*_t_ by 6%, and *d*_33_ by 27%. At the same time, they adopted a structure of two single crystals connected in parallel, further enhancing the capacitance of the transducer and achieving better impedance matching, resulting in an unprecedented 224% increase in the receiver voltage response of the 200 kHz transducer [116]. Zheng et al. developed a 6 MHz cardiac internal ultrasound echocardiography probe based on 23PIN-45PMN-32PT single crystals. The bandwidth improvement of the transducer made from ACP samples was very small, but the sensitivity increased by 20%, demonstrating deeper penetration depth. The results showed that ACP had more advantages in miniaturized devices [117]. Additionally, Kim et al. proved that low-frequency alternating electric fields could completely depolarize and re-polarize the assembled PIN-PMN-PT transducers at room temperature without causing a decline in device performance. Therefore, this method provided a feasible alternative to the traditional thermal depolarization method and could precisely control the excitation uniformity of phased array transducer elements. This discovery provided a new strategy for changing the polarization state of ferroelectric materials and offered the possibility of regulating the consistency of array elements in phased arrays [34]. Zhu et al. processed [001]-oriented PMN-PT single crystals using the ultra-high temperature field cooling (UFCP) method. Compared with traditional DCP, *d*_33_ increased by 51% and *Q*_m_ increased by 23%. The ultrasonic transducer with thickness vibration mode made from this optimized single crystal showed a 7.2% −6 dB bandwidth increase [122]. Besides acoustic imaging applications, Chen et al. also applied ACP to PMN-PT materials in bidirectional push-pull magneto-electric sensors, significantly suppressing magnetic noise and significantly improving the final detection sensitivity [123].

The above research indicates that the ACP method has gradually shifted from performance optimization at the material level to integrated innovation at the transducer system level, and is expected to provide an effective solution for resolving the core contradiction among bandwidth, sensitivity and array density in current medical ultrasound imaging.

## 5. Overall Conclusions and Future Outlook

The comprehensive review of the literature from 2020 to 2026 indisputably establishes Alternating Current Poling (ACP) as a transformative domain engineering platform that fundamentally alters the performance trajectory of medical ultrasound transducers. Historically, transducer design was severely constrained by the physical trade-off between axial resolution (dictated by bandwidth) and penetration depth (dictated by sensitivity). ACP disrupts this dichotomy by restructuring the microscopic thermodynamic energy landscape of piezoelectric materials—such as PMN-PT, PIN-PMN-PT, and PZT composites—inducing highly uniform, dense 109° stripe domains that minimize internal mechanical losses and maximize electromechanical energy conversion.

The transition of ACP from theoretical material science to practical acoustic engineering has yielded extraordinary device-level breakthroughs. From the suppression of lateral crosstalk in phased arrays to the achievement of superhigh bandwidths (exceeding 140%) in piezocomposites, ACP has consistently outperformed traditional Direct Current Poling (DCP). Furthermore, researchers have successfully mitigated the mechanical degradation inherent in array dicing via hybrid ACP-DCP protocols, and have pioneered room-temperature in-device repoling to extend transducer lifecycles. Recent advancements have leveraged structural synergies—coupling ACP crystals with acoustic reflection layers, stacked parallel architectures, and ultrahigh-temperature field cooling (UFCP)—producing massive, non-linear enhancements in sensitivity (up to 224%) and pressure output (up to 66%). Ultimately, ACP provides a highly cost-effective, easily implementable pathway to next-generation high-fidelity medical imaging without the need for complex chemical doping. Due to the advantages of ACP in enhancing the performance of single crystals, the application prospects of high-end medical equipment based on single crystals are very broad. The application of ACP in ultrasonic transducers is expected to expand to highly specialized and extreme environment fields, such as intravascular imaging and long-term continuous biological monitoring.

In conclusion, while alternating current poling (ACP) represents a transformative advancement in amplifying the electromechanical parameters of relaxor-PT single crystals, the transition towards clinical implementation necessitates resolving several critical challenges. First, batch-to-batch reproducibility across different single-crystal boules grown by the Bridgman method remains a key obstacle, as spatial compositional bias (e.g., Ti-segregation) readily shifts the narrow optimal ACP parameter window. Second, the long-term aging stability of the structurally softened, ACP-induced metastable phases under continuous clinical operational stress—encompassing continuous high-voltage therapeutic pulses, ultrasonic driving, and sustained core body temperatures—requires systemic and rigorous experimental validation to prevent unexpected in-use depolarization. Addressing these manufacturing and reliability challenges will define the next phase of ACP research.

## Data Availability

Data are contained within the article.

## Supplementary Materials

The following supporting information can be downloaded at: https://www.mdpi.com/article/10.3390/s26134292/s1, Table S1: Comparative statistics of PMN-PT single crystal performance under ACP and DCP; Table S2: Comparative statistics of PIN-PMN-PT single crystal performance under ACP and DCP; Table S3: Comparative statistics of Mn-doped PIN-PMN-PT single crystal performance under ACP and DCP; Table S4: Comparative statistics of other single crystals’ performance under ACP and DCP. The data were extracted from papers published between 2018 and 2025. Table S5: Comparison of the performance of transducers under ACP and DCP. The data were extracted from papers published between 2020 and 2025.
