# Supplementary material for: Optimization of Piezoelectric Materials and Ultrasound Imaging Transducers via Alternating Current Poling
Source: Sensors (Basel). 2026 Jul 6;26(13):4292. doi: 10.3390/s26134292 (PMC13364390; doi:10.3390/s26134292)
Supplement: Supplementary file 1 [file sensors-26-04292-s001.zip › sensors-4372163-supplementary.pdf]

Table S1. Comparative statistics of PMN-PT single crystal performance under ACP and DCP. The data were extracted from papers published between 2018 and 2025.

Year	Comps.	C.O.	Amp. (kV/cm)	*f* (Hz)	*N* (cycles)	*ε*-DCP	*ε*-ACP	*ε*-I.R. (%)	d_{33}-DCP (pC/N)	d_{33}-ACP (pC/N)	*d*-I.R. (%)	*k*-DCP	*k*-ACP	*k*-I.R. (%)	Ref.
2018	PMN-25PT	[001]	12–18 (V_p)	20–40	20	5321	6397	20.22%	1220	1730	41.80%	~56%(k_t)	~56%(k_t)	/	[13]
2018	PMN-30PT	[001]	10.00 (V_{pp})	1	7	6120	8500	38.89%	1650	2000	21.21%	57%(k_t)	57%(k_t)	/	[48]
2019	PMN-30PT	[001]	10.00 (V_{pp})	0.01	5	6287	10,500	67.01%	1916	3200	67.01%	92%(k_{33})	94%(k_{33})	2%	[40]
2019	PMN-30PT	[001]	10.00 (V_{pp})	1	7	5090	7570	48.72%	1380	1930	39.86%	58%(k_t)	60%(k_t)	2%	[33]
2019	PMN-30PT	[001]	15.00 (V_p)	3	20	4620	6250	35.28%	1380	1860	34.78%	/	/	/	[50]
2019	PMN-30PT	[100]	12.00 (V_{pp})	20	40	8490	8800	3.65%	2710	3050	12.55%	95%(k_{33})	94%(k_{33})	−1%	[51]
2019	PMN-29PT	[001]	1.00 (V_p)	1	10	5291	6586	24.48%	1352	1688	24.85%	/	/	/	[31]
2020	PMN-28PT	[001]	2.50 (S-V_{rms})	0.1	10	6330	9560	51.03%	1780	2470	38.76%	58%(k_t)	58%(k_t)	/	[52]
2020	PMN-28PT	[001]	5.00 (V_p)	10	10	5800	7800	34.48%	1670	2190	31.14%	93%(k_{33})	94%(k_{33})	1%	[14]
2020	PMN-30PT	[001]	3.00 (S-V_{rms})	0.01	6	6110	7920	29.62%	1690	2290	35.50%	93%(k_{33})	94%(k_{33})	1%	[53]
2021	PMN-27PT	[001]	2.89 (T-V_{rms})	0.1	10	5300	7220	36.23%	1410	1920	36.17%	89%(k_{33})	92%(k_{33})	3%	[47]
2021	PMN-26PT	[001]	5.00 (V_p)	1	10	5100	6200	21.57%	1400	1900	35.71%	/	/	/	[15]
2021	PMN-28PT	[001]	3.00 (S-V_{rms})	0.1	10	5940	7270	22.39%	1350	1830	35.56%	58%(k_t)	58.30%(k_t)	0.3%	[10]
2022	PMN-28PT	[001]	3.50 (S-V_{rms})	0.1	10	5490	7000	27.50%	1350	1570	16.30%	57.30%(k_t)	57.70%(k_t)	0.4%	[54]
2022	PMN-26PT	[001]	20.00 (V_p)	0.1	5	4610	6600	43.17%	1340	1820	35.82%	53.90%(k_t)	56.80%(k_t)	2.9%	[32]
2022	PMN-28PT	[001]	5.00 (T-V_{rms})	5	20	5680	7970	40.32%	1660	2000	20.48%	62%(k_t)	65%(k_t)	3%	[55]
2022	PMN-30PT	[001]	3.00 (S-V_{rms})	0.1	12	6000	9690	61.50%	1650	2920	76.97%	59.40%(k_t)	59.80%(k_t)	0.4%	[49]
2022	PMN-30PT	[001]	3.00 (S-V_{rms})	0.1	12	6040	10,270	70.03%	1720	2620	52.33%	92.80%(k_{33})	95.10%(k_{33})	2.3%	[16]
2022	PMN-30.5PT	[001]	8.00 (S-V_{rms})	50	50	9700	14,500	49.48%	2750	4200	52.73%	59.30%(k_t)	59.50%(k_t)	0.2%	[35]
2023	PMN-(30–33)PT	[001]	10.00 (V_p)	1	40	/	/	/	1430	2400	67.83%	/	/	/	[18]
2023	PMN-30PT	[001]	5.00 (S-V_{rms})	/	/	6150	11,200	82.11%	1640	3180	93.90%	60.40%	59.20%	−1.2%	[56]

Comps.: Sample Components; CO: Crystallographic Orientation; Amp.: ACP *E*-field Amplitude (V_p: peak amplitude value; V_{pp}: peak-peak amplitude value; S: Sine-wave voltage; T: Triangle-wave voltage; V_{rms}: root mean square value of AC signals); *f*: ACP *E*-field frequency; *N*: Number of ACP *E*-field cycles; I.R. (%): Improvement (%); *ε* represents $\varepsilon_{33}^{T}/\varepsilon_0$.

Table S1. Comparative statistics of PMN-PT single crystal performance under ACP and DCP. The data were extracted from papers published between 2018 and 2025.

Year	Comps.	C.O.	Amp. (kV/cm)	f (Hz)	N (cycles)	ε-DCP	ε-ACP	ε-I.R. (%)	d_{33}-DCP (pC/N)	d_{33}-ACP (pC/N)	d-I.R. (%)	k-DCP	k-ACP	k-I.R. (%)	Ref.
2023	PMN-30PT:Yb	[001]	14.00 (V_p)	15	10	/	/	/	1900	2670	40.53%	/	/	/	[57]
2023	PMN-29PT	[001]	5.87 (T-V_{rms})	1	20	6320	7150	13.13%	1520	1760	15.79%	/	/	/	58]
2024	PMN-PT(MPB)	/	12.00 (V_{pp})	20	1–14	/	/	/	/	/	/	/	/	/	[59]
2024	PMN-PT:Sm	[001]	7.59 (V_p)	0.5	35	10,260	13,290	29.53%	3630	4520	24.52%	/	/	/	[34]
2024	PMN-PT	[001]	8.00 (V_p)	1	15	4100–5000	6500–7600	/	1200–1300	2100–2500	/	/	/	/	[60]
2024	PMN-30PT	[001]	3.00 (S-V_{rms})	0.1	12	6200	11,000	77.42%	1800	3000	66.67%	59.50%(k_t)	60.50%(k_t)	1%	[61]
2024	PMN-26 PT	[001]	14.00 (V_p)	0.1	5	5145	5881	14.31%	1250	1780	42.40%	52%	58%	6%	[62]
2025	PMN-30PT	[001]	15.00 (V_{pp})	0.1	10	/	/	/	668(d_{31})	1126(d_{31})	68.60%	~42%(k_{31})	56%(k_{31})	14%	[63]
2025	PMN-29PT	[001]	10.00 (V_{pp})	1	20	/	/	/	/	/	/	/	/	/	[64]
2025	PMN-PT	[001]	12.00 (V_{pp})	1	10	~6000	8200	~36.67%	1655	2480	49.85%	/	/	/	[65]
2025	PMN-PT:Yb	[001]	7.00 (V_p)	10	20	~3200	~4000	~25%	1000	1650	65.00%	/	/	/	[66]
2025	PMN-28PT	[001]	10.00 (V_{pp})	1	0–50	/	/	/	/	/	/	/	/	/	[67]
2025	PMN-30.5PT	[001]	3.00 (S-V_{rms})	1	80	10,140	14,700	45%	3210	4200	30.84%	59.70%(k_t)	61.50%(k_t)	1.8%	[38]
2025	PMN-30PT	[001]	3.00 (S-V_{rms})	1	18	6150	11,200	82%	1640	3190	94.51%	60%(k_t)	59%(k_t)	−1%	[39]
2025	PMN-26PT	[001]	3.00 (S-V_{rms})	1	18	5190	6390	23%	1350	1760	30.37%	58%	59%	1%	[39]
2025	PMN-29.5PT	[001]	3.00 (S-V_{rms})	1	18	6530	9150	40%	1750	2800	60%	57%	60%	3%	[39]
2025	PMN-30.5PT	[001]	3.00 (S-V_{rms})	1	18	10,500	14,000	33%	3200	4000	25%	63%	62%	−1%	[39]
2025	PMN-33PT		3.00 (S-V_{rms})	1	18	4800	6000	25%	1660	1820	9.6%	61%	63%	2%	[39]
2025	PMN-29PT	[001]	10.00 (V_{pp})	1	3	/	/	/	/	/	/	/	/	/	[68]
2025	PMN-30PT	[001]	14.00 (V_{pp})	1	23	/	/	21.62%	/	/	45.67%	/	/	/	[21]

Comps.: Sample Components; CO: Crystallographic Orientation; Amp.: ACP E-field Amplitude (V_p: peak amplitude value; V_{pp}: peak-peak amplitude value; S: Sine-wave voltage; T: Triangle-wave voltage; V_{rms}: root mean square value of AC signals); f: ACP E-field frequency; N: Number of ACP E-field cycles; I.R. (%): Improvement (%); ε represents $\varepsilon_{33}^{T}/\varepsilon_0$.

Table S2. Comparative statistics of PIN-PMN-PT single crystal performance under ACP and DCP. The data were extracted from papers published between 2018 and 2025.

Year	Comps.	C.O.	Amp. (kV/cm)	f (Hz)	N (cycles)	ε-DCP	ε-ACP	ε-I.R. (%)	d_{33}-DCP (pC/N)	d_{33}-ACP (pC/N)	d-I.R. (%)	k-DCP	k-ACP	k-I.R. (%)	Ref.
2020	20PIN-39PMN-41PT	[001]	14.00 (V_p)	10	4	806	814	0.99%	330	319	−3.33%	/	/	/	[69]
2020	20PIN-39PMN-41PT	[110]	16.00 (V_p)	10	4	3430	4320	25.95%	630	831	31.90%	/	/	/	[69]
2020	31PIN-43PMN-26PT	[001]	8.00 (S-V_{rms})	0.1	20	3280	3290	0.30%	1000	1000	/	87.30%(k_{33})	85.50%(k_{33})	−1.8%	[53]
2020	23PIN-47PMN-30PT	[001]	10.00 (V_p)	1	10	5533	6231	12.62%	1624	2020	24.38%	/	/	/	[70]
2020	23PIN-47PMN-30PT	[011]	10.00 (V_p)	1	10	~3000	~3000	/	~900	~900	/	/	/	/	[70]
2020	22PIN-44PMN-34PT	[001]	10.00 (V_p)	1	10	~4100	~3600	−0.12%	~1800	~1580	−12.2%	/	/	/	[70]
2020	25PIN-43PMN-32PT	[001]	10.00 (V_p)	50	20	4800	7120	48.33%	1700	2610	53.53%	/	/	/	[70]
2021	PIN-PMN-PT (R)	[001]	7-10 (V_p)	0.1–1	12–40	/	/	/	1730	2530	46.24%	/	/	/	[71]
2021	25PIN-42PMN-33PT	[001]	10.00 (V_p)	50	20	5080	7440	46.46%	–870 (d_{31})	−1560 (d_{31})	79.31%	83% (k_{31})	92% (k_{31})	9%	[24]
2021	PIN-PMN-PT (MPB)	[001]	10.00 (V_p)	50	20	5258	8224	56.40%	−961 (d_{31})	−1663 (d_{31})	62.16%	/	/	/	[72]
2021	24PIN-46PMN-30PT	[001]	4.00 (S-V_{rms})	10	12	5440	7000	29.00%	1810	2340	29.28%	93.80%(k_{33})	94.80%(k_{33})	1%	[73]
2022	PIN-PMN-PT	[001]	8.90 (T-V_{rms})	~35	20	4080	5410	32.60%	1180	1530	29.66%	/	/	/	[74]
2022	27PIN-46PMN-27PT	[001]	15.00 (V_p)	40	10	3663	4379	19.55%	1148	1373	19.60%	/	50%	/	[17]
2022	24PIN-46PMN-30PT	[001]	4.00 (S-V_{rms})	0.1	20	7000	8330	19.00%	2340	2750	17.52%	59.30%(k_t)	58.20%(k_t)	−1.1%	[37]
2022	24.5PIN-PMN-35PT	[001]	10.00 (V_{pp})	50	20	5914	7710	30.37%	2150	2574	19.72%	/	/	/	[75]
2022	PIN-PMN-30PT	[001]	8.00 (S-V_{rms})	50	50	5140	8930	73.74%	1680	2620	55.95%	93.70%(k_{33})	95.6%(k_{33})	1.9%	[16]
2022	PIN-PMN-26PT	[001]	8.00 (S-V_{rms})	50	100	3260	3620	11.04%	990	1050	6.06%	88%(k_{33})	88.70%(k_{33})	0.7%	[16]

Comps.: Sample Components; CO: Crystallographic Orientation; Amp.: ACP E-field Amplitude (V_p: peak amplitude value; V_{pp}: peak-peak amplitude value; S: Sine-wave voltage; T: Triangle-

wave voltage; V_{rms}: root mean square value of AC signals); f: ACP E-field frequency; N: Number of ACP E-field cycles; I.R. (%): Improvement (%); ε represents $\varepsilon_{33}^{T}/\varepsilon_{0}$.

Table S2. Comparative statistics of PIN-PMN-PT single crystal performance under ACP and DCP. The data were extracted from papers published between 2018 and 2025.

Year	Comps.	C.O.	Amp. (kV/cm)	f (Hz)	N (cycles)	ε-DCP	ε-ACP	ε-I.R. (%)	d_{33}-DCP (pC/N)	d_{33}-ACP (pC/N)	d-I.R. (%)	k-DCP	k-ACP	k-I.R. (%)	Ref.
2022	22PIN-53PMN-25PT	[001]	10.00 (V_p)	10	~20	/	/	/	/	~3100	/	/	~52%(k_t)	/	[27]
2022	25PIN-43PMN-32PT	[001]	10.00 (V_p)	50	20	4746	6469	36.30%	−798 (d_{31})	−1294 (d_{31})	62.16%	/	/	/	[30]
2023	PIN-PMN-PT	[001]	20.00 (V_{pp})	5	20	3407	4593	34.81%	1210	1420	17.36%	54%	53%	−1%	[76]
2023	PIN-PMN-PT	[001]	6-8 (S-V_{rms})	0.1	15	3410	4290	25.81%	1200	1500	25.00%	/	/	/	[77]
2023	24PIN-46PMN-30PT	[001]	3.58 (S-V_{rms})	0.1	1–22	5500	7500	36.36%	1800	2300	27.78%	/	/	/	[78]
2024	24PIN-44PMN-32PT	[001]	5.00 (V_p)	1	3	/	/	/	~1500	~2400	56.00%	/	/	/	[79]
2024	PIN-PMN-PT	[011]	8.00 (V_p)	1	3	/	/	/	~1300	~650	-53%	/	/	/	[79]
2024	PIN-PMN-PT	[001]	5.80 (T-V_{rms})	30	20	4630	6170	33.26%	1400	1820	30.00%	/	/	/	[34]
2025	23PIN-47PMN-30PT	[001]	10.00 (V_p)	1	25	3246	3819	17.65%	1076	1270	18.03%	/	/	/	[29]
2025	23PIN-47PMN-30PT	[011]	10.00 (V_p)	1	25	1671	1523	−8.86%	707	667	−5.66%	/	/	/	[29]
2025	23PIN-47PMN-30PT	[111]	10.00 (V_p)	1	25	546	764	39.93%	78	45	−42%	/	/	/	[29]
2025	PIN-PMN-PT:Sm	[001]	/	/	/	5940	6200	4.38%	1320	1540	16.67%	44.00%	67.00%	23%	[80]
2025	27PIN-46PMN-27PT	[011]	15.00 (V_p)	100	10	2394	2780	16.12%	637	676	6.12%	/	42.00%	/	[36]
2025	15PIN-60PMN-25PT	[001]	8.00 (V_{pp})	0.1	10	/	/	/	1424	1985	39.40%	54.90%(k_t)	57.20%(k_t)	−2.3%	[81]

Comps.: Sample Components; CO: Crystallographic Orientation; Amp.: ACP E-field Amplitude (V_p: peak amplitude value; V_{pp}: peak-peak amplitude value; S: Sine-wave voltage; T: Triangle-wave voltage; V_{rms}: root mean square value of AC signals); f: ACP E-field frequency; N: Number of ACP E-field cycles; I.R. (%): Improvement (%); ε represents $\varepsilon_{33}^{T}/\varepsilon_0$.

Table S3. Comparative statistics of Mn-doped PIN-PMN-PT single crystal performance under ACP and DCP. The data were extracted from papers published between 2018 and 2025.

Year	Comps.	C.O.	Amp. (kV/cm)	f (Hz)	N (cycles)	ε-DCP	ε-ACP	d_{33}-DCP (pC/N)	d_{33}-ACP (pC/N)	k-DCP	k-ACP	Q_m-DCP	Q_m-ACP	Ref.
2020	32PIN-36PMN-32PT: Mn-0.5 mol%	[001]	10.00 (V_{pp})	1	20	4879	5997	1200	1380	55%(k_t)	63%(k_t)	/	/	[11]
2021	PIN-PMN-27PT: Mn-1 mol%	[001]	20 (V_p)	0.1	20	3700	5300	1350	1750	90.8%(k_{33})	91.5%(k_{33})	660(Q_{33})	770(Q_{33})	[82]
2023	27PIN-46PMN-27PT:Mn	[001]	15 (V_p)	25	10	2387	2694	1015	1174	/	/	153(Q_{31})	231(Q_{31})	[42]
2024	PIN-PMN-PT:Mn (MPB)	[001]	8 (V_p)	1	8	/	/	1435	2194	/	/	193(Q_{31})	420(Q_{31})	[43]
2025	PIN-PMN-PT:Mn-1 mol%	[001]	/	/	/	3800	4800	1400	1750	44%(k_t)	50%(k_t)	740	700	[80]
2025	PIN-PMN-32PT:Mn	[001]	10 (V_p)	1	10	3340	6150	1420	2280	/	/	/	700	[41]
2025	25PIN-46PMN-29PT:Mn	[001]	10-15 (V_p)	1–10	10–20	2060	3550	1550	1880	/	/	/	/	[28]

Comps.: Sample Components; CO: Crystallographic Orientation; Amp.: ACP E-field Amplitude (V_p: peak amplitude value; V_{pp}: peak-peak amplitude value; S: Sine-wave voltage; T: Triangle-wave voltage; V_{rms}: root mean square value of AC signals); f: ACP E-field frequency; N: Number of ACP E-field cycles; I.R. (%): Improvement (%); ε represents $\varepsilon_{33}^T/\varepsilon_0$.

Table S4. Comparative statistics of other single crystals' performance under ACP and DCP. The data were extracted from papers published between 2018 and 2025.

Year	Comps.	C.O.	Amp. (kV/cm)	f (Hz)	N (cycles)	ε-DCP	ε-ACP	ε-I.R. (%)	d_{33}-DCP (pC/N)	d_{33}-ACP (pC/N)	d-I.R. (%)	k-DCP	k-ACP	k-I.R. (%)	Ref.
2019	52PMN-15PYbN-33PT	[001]	11.00 (V_p)	10	20	5200	6800	30.77%	1770	2490	40.68%	57%(k_t)	57%(k_t)	/	[44]
2021	51PMN-11PYbN-38PT	[001]	8.00 (V_p)	10	20	3670	4990	35.97%	790	1040	31.65%	/	/	/	[26]
2021	66 PIN-34 PT	[001]	11.60 (V_p)	15	20–25	2690	3070	14.13%	1180	1400	18.64%	66%(k_{33})	86%(k_{33})	20%	[45]
2022	65.5PIN-34.5PT	[001]	15.00 (V_p)	5	10-30	2970	3690	24.24%	1390	1720	23.74%	/	/	/	[19]
2022	65PIN-35PT	[001]	20.00 (V_p)	1–5	10-30	3620	3360	−7.18%	1640	1490	−9.15%	/	/	/	[46]
2023	PMN-30PZT:La	[001]	6.00 (T-V_{rms})	1	10	10,000	14,200	42.00%	2800	4800	71.43%	47%(k_t)	48.2%(k_t)	1.2%	[88]
2023	NKBT:Mn	[001]	20.00 (V_p)	1	25	/	/	/	135	155	14.81%	/	/	/	[83]
2024	PZN-5.5PT	[001]	6.00 (S-V_{rms})	2	7	5610	6680	19.07%	2100	2760	31.43%	45.9%(k_t)	42.6%(k_t)	−3.3%	[84]
2024	40PMN-25PZ-35PT: 0.5MnO_2	[001]	6.80 (T-V_{rms})	1	600	3990	5962	49.42%	1345	2024	50.48%	/	/	/	[85]
2025	Mn&Fe: KTN	[100]	8	1	24	/	/	/	~310	~310	/	/	/	/	[86]

Comps.: Sample Components; CO: Crystallographic Orientation; Amp.: ACP E-field Amplitude (V_p: peak amplitude value; V_{pp}: peak-peak amplitude value; S: Sine-wave voltage; T: Triangle-wave voltage; V_{rms}: root mean square value of AC signals); f: ACP E-field frequency; N: Number of ACP E-field cycles; I.R. (%): Improvement (%); ε represents $\varepsilon_{33}^{T}/\varepsilon_0$.

Table S5. Comparison of the performance of transducers under ACP and DCP. The data were extracted from papers published between 2020 and 2025.

Year	Piezoelectric material	Frequency	-6dB Bandwidth (%) (DCP)	Sensitivity (DCP)	-6dB Bandwidth (%) (ACP)	Sensitivity (ACP)	Ref.
2020	PMN-PT S.C.	10 MHz	47.9	21.4(mV/V)	58.2	24.4(mV/V)	[110]
2020	PMN-PT S.C.	~2.5 MHz	79.21	-34.85 dB	86.41	-34.06 dB	[112]
2022	1-3 PZT-5H Ce.	3 MHz	89.8	-18.8 dB	112.1	-17.6 dB	[108]
2022	1-3 PMNT S.C.	3 MHz	107.9	-17.0 dB	142.8	-16.1 dB	[108]
2023	2-2 PZT-5H Ce.	3 MHz	64.52	-48 dB	78.78	-22 dB	[118]
2023	1-3 PMN-PT S.C.	3 MHz	50.08	-37.71 dB	56.35(fc=2.52 MHz)	-35.78 dB(fc=2.52 MHz)	[113]
2023	PIN-PMN-PT S.C.	10 MHz	44.31	-38.64 dB	55.04	-35.34 dB	[23]
2024	PIN-PMN-PT S.C.	~2.8 MHz	79.77	-39.80 dB	86.46	-39.01 dB	[119]
2024	PMN-PT S.C.	~200 kHz	59.8	21.6 mV	63.6	26.7mV	[116]
2025	2-2 PIMNT S.C.	~2.1 MHz	56.04	-30.20 dB	63.98	-27.78 dB	[114]
2025	PMN-PT S.C.	~2.6 MHz	78.02	-54.42 dB	84.39	-53.15 dB	[115]
2025	PIN-PMN-PT S.C.	~6 MHz	88	2.6 V	91	3.11 V	[117]
2025	PMN-PT S.C.	~10 MHz	46.8	1.48 V	52.4	1.74 V	[111]

S.C.: Single crystal; Ce.: Ceramic.
